# Resilient frequency stabilization of renewable-penetrated microgrids via deep learning and machine learning

**DOI:** 10.1038/s41598-026-51207-5

**Published:** 2026-05-08

**Authors:** Jasmine Hansda, Prakash K. Ray, Asit Mohanty, Soumya Ranjan Das, Erdem Cuce

**Affiliations:** 1 School of Electrical Sciences, OUTR, Bhubaneswar, Odisha India; 2 Institute of Power Engineering, National Energy University, Kualalumpur, 43000 Malaysia; 3 Centre for Promotion of Research, Graphic Era (Deemed to be University), Clement town, Dehradun, India; 4 Manipal Institute of Technology, Manipal Academy of Higher Education, Manipal, India; 5 Department of Mechanical Engineering, Faculty of Engineering and Architecture, Recep Tayyip Erdogan University, Zihni Derin Campus, Rize, 53100 Türkiye; 6 University Centre for Research and Development, Chandigarh University, Mohali, 140413 Punjab India

**Keywords:** Deep neural network (DNN), Grey wolf optimization (GWO), Micro grid system, Particle swarm optimization (PSO), Support vector regression (SVR), Wind energy system (WES), Energy science and technology, Engineering, Mathematics and computing

## Abstract

The intrinsic variability of wind generation necessitates accurate wind speed forecasting to minimize active power imbalance between supply and demand and thereby enhance frequency stability in microgrids (MGs). In this context, this paper investigates wind speed prediction at four geographically distinct sites using Support Vector Regression (SVR) and Deep Neural Networks (DNN) and analyses their impact on microgrid (MG) frequency regulation. The predicted wind data are incorporated as disturbances in a MG system comprising Wind Turbine generator (WTG), Diesel Engine Generator (DEG), Aqua-Electrolyser (AE), Fuel Cell (FC), Battery Energy Storage System (BESS), Flywheel Energy Storage System (FESS), and Ultra Capacitor (UC) units. To mitigate frequency deviations caused by load variations and wind power uncertainty, a PID controller optimized using a hybrid Particle Swarm Optimization (PSO) and Grey Wolf Optimization (GWO) algorithm is proposed. The forecasting analysis indicates that the SVR model outperforms the DNN model, reducing prediction errors by up to 52.94% in MAE, 44.59% in MAPE, 83.54% in MSE, and 59.55% in RMSE. Simulation results further demonstrate that the hybrid PSO–GWO tuned PID controller significantly improves dynamic performance compared with PSO-PID and GWO-PID controllers. For the SVR-based prediction case, the proposed controller reduces maximum overshoot (Mp) by 50%, settling time (Ts) by 16.13%, and integral square error (ISE) by 77.78% compared with PSO, while achieving 33.33%, 10.86%, and 50% improvements over GWO, respectively. Similarly, for the DNN-based case, the hybrid controller achieves further improvements of 92.5% in Mp, 21.21% in Ts, and 57.14% in ISE compared with PSO. The proposed approach is validated through time- and frequency-domain analyses and real-time implementation using the OPAL-RT Hardware-in-the-Loop (HIL) platform, confirming its effectiveness in enhancing the stability and reliability of renewable-integrated MGs.

## Introduction

Various ecological factors such as global warming, pollution, and rapid depletion of conventional fossil fuels like coal have driven growth for Renewable Energy Sources (RES) such as wind, solar, tidal, mini-hydro etc. Among these renewable sources Wind Energy Systems (WES) has become forefront of energy generation due to rapid improvements in technological advances in design of wind turbines, generators and power electronics-based interfacing converters^1,2^. However, the integration of wind energy into power systems remains challenging due to its inherent intermittency and variability, which can adversely affect system performance, leading to stability issues and frequency fluctuations. Hence, to minimize the fluctuations these resources are being integrated with diesel engine generator (DEG), fuel cell (FC) and energy storage (ES) options like battery energy storage system (BESS), flywheel energy storage system (FESS) and Ultracapacitor (UC) to formulate a micro grid^3^. Alternatively, the uncertainties in the power output can be managed by accurately predicting the input wind speed by some efficient techniques. This, not only helps to understand the impact of intermittency and power available from wind energy system to the MG before time, but also helps in committing the conventional resources, diesel generators, fuel cells and the ES options to manage the energy demands^4,5^.

In literature, various methods for wind speed prediction are proposed by different researchers. Numerical Weather Prediction (NWP) model is employed for long term forecasting and requires enormous computation speed to run and are mainly employed in meteorological centres for yearly and seasonal predictions^6^. Statistical approaches such as Auto-Regressive Moving Average (ARMA), Auto-Regressive Integrated Moving Average (ARIMA)^7^ are used for wind speed prediction where the historical data is used to frame a model for future predictions^8,9^. Similarly, another method called persistence technique^10,11^ is applied for shorter term wind speed prediction where it is assumed that wind speed at time ‘*t+∆t’* is same as that at ‘*t*’. In recent years, development of new techniques like Artificial Neural Network (ANN)^12^, Wavelet Transform, Spatial Correlation and Fuzzy Logic Control have been introduced for future data predictions^13–15^. Again, different combinations of classical techniques with modern methods are used to formulate hybrid techniques for short term predictions producing accurate result^16^. Among these techniques combination of NWP and NN, Adaptive Neuro-Fuzzy Inference (ANFIS) are proving to be extremely capable to handle stochastic predictions^17,18^. But, each of these methods suffer from some disadvantages such as NWP can efficient for long term prediction and require supercomputers and ANN based models are effective for short term predictions^19^. Therefore, machine learning techniques such as Support Vector Machines (SVM) can be effectively utilized for regression problems due to their strong generalization capability and superior performance compared with several conventional machine learning methods^20,21^. In^22^, Support Vector Regression (SVR) was employed to construct a model that fits the observed wind speed and wind power data. The Particle Swarm Optimization (PSO) algorithm was used to optimize key model parameters, including the penalty factor (C), precision parameter (ε), and kernel function variance (σ). In another study, a machine learning analysis based on the Random Forest algorithm was performed using a Kaggle dataset to forecast the future potential of renewable energy, achieving high prediction accuracy and low error rates^23^. Furthermore, the CNN–Attention–GRU model was proposed in^24^ for accurate forecasting, where an attention mechanism is integrated with CNN–GRU to reduce the impact of weather feature variability. In addition, Deep Learning (DL) has emerged as a powerful supervised learning approach in which models are constructed using multiple layers to extract high-level abstract features from data^25^. DL models utilize the backpropagation algorithm to capture complex data patterns, and the use of Recurrent Neural Networks (RNNs) within deep learning frameworks has proven effective for various supervised learning tasks^26^. Moreover, Author^27^ proposed a model for short-term load forecasting using Deep Neural Networks (DNN), demonstrating that DNNs can effectively learn complex relationships between input and output sequences.

Thus, these methods are proposed in this paper for wind speed predictions. Real-time wind speed data at four different sites are taken in this study for future prediction of wind speed using SVR and DNN. In addition, the predicted wind speed and corresponding wind power output are considered as input disturbances to the studied MG system^28^, which consists of WTG, DEG, AE, FC, and energy storage systems such as BESS, FESS, and UC. This setup is used to analyze their impact on system frequency deviations. Load frequency control (LFC) becomes a critical issue in such systems due to the imbalance between power generation and load demand^29–31^. In this context, designing an efficient controller to mitigate system frequency deviations is essential. Several advanced control strategies have been proposed, including adaptive control, artificial neural network control, variable structure control, sliding mode control, fuzzy logic control, and model predictive control. Although these approaches provide good performance in dynamic systems, they often suffer from drawbacks such as complex design procedures, high computational burden, dependence on designer expertise for parameter tuning, and higher implementation costs. In contrast, traditional controllers are simple and inexpensive but face challenges in handling system nonlinearities and parameter variations. Among them, proportional-integral-derivative (PID) controllers^32–35^ is widely used due to their simple structure, low cost, and satisfactory stability margin. To improve their performance, meta-heuristic optimization algorithms are commonly employed to optimally tune the controller gains.

Numerous meta-heuristic optimization techniques have been widely applied to address the LFC. These include algorithms such as the Mountaineering Team-Based Optimization (MTBO) algorithm^29^, Marine Predators Algorithm (MPA)^30^, Differential Evolution (DE), and Firefly Algorithm (FA)^31^. In addition, several optimized control strategies have been proposed, including Differential Evolution–tuned PID controllers^32^, Repulsive Firefly Algorithm-based PID controllers^33^, and Walrus Optimization-based adaptive virtual inertia control with PID controllers^35^. Other studies have utilized Particle Swarm Optimization (PSO)-tuned PID controllers^36^, the Dragonfly Algorithm^37^, and the Squid Game Optimizer (SGO)-based fractional-order proportional–integral double-derivative controllers^38^. Furthermore, advanced controller designs such as quasi-oppositional-based Whale Optimization Algorithm (WOA)-tuned PID, 3DOF-PID, and fuzzy-3DOF-PID controllers^39^, and hybrid Grasshopper Optimization Algorithm–PSO tuned PID controllers^40^ have also been reported to improve LFC performance. Among various optimization methods, PSO and the Grey Wolf Optimizer (GWO**)**^41^ have attracted significant attention due to their complementary exploration and exploitation capabilities. PSO effectively explores the search space through particle interactions, whereas GWO efficiently exploits promising solutions using its leadership-based hunting mechanism. However, PSO may suffer from premature convergence and stagnation in local optima, while GWO may exhibit limited exploration ability in complex search spaces. To overcome these limitations, a hybrid PSO–GWO algorithm is employed to achieve a better balance between exploration and exploitation, thereby improving optimization performance. For instance, Author^42^ developed a GWO-PSO-PID controller for a pump–motor servo system, achieving improved rise time, robustness, and noise immunity. Similarly, Author^43^ applied a hybrid PSO–GWO approach for robust feedback control of nonlinear systems, demonstrating faster convergence and fewer cost function evaluations compared with standalone optimization methods. In addition, Author^44^ utilized a hybrid PSO–GWO optimized PID controller for Load Frequency Control (LFC) in standalone marine MGs. These studies highlight the effectiveness of the hybrid PSO–GWO approach in enhancing convergence speed and improving solution quality for controller parameter optimization.

### Research gap identified from literature

Based on the literature survey, several key observations are identified as follows.

1. Most existing wind speed prediction studies rely on large historical datasets spanning several years to train machine learning or deep learning models^14,16,19^. However, in many practical scenarios, only limited historical data may be available. Therefore, there is a need to investigate the effectiveness of prediction models such as DNN and SVR when trained with short-duration datasets.
2. Many studies evaluate prediction models using data from a single location or Two locations^6,21,45^, which may not represent varying wind characteristics. Therefore, evaluating prediction models across multiple geographical locations is necessary to verify their robustness and generalization capability.
3. Most wind speed prediction studies focus primarily on forecast accuracy, while their impact on power system applications such as LFC has not been widely explored^28^. Furthermore, many existing LFC studies in MG systems use assumed or artificial disturbance signals^30,31,34,44^. Only a few studies incorporate predicted wind speed or wind power data from multiple real locations to analyze their impact on the frequency stability of MG containing renewable sources and storage devices.
4. Many studies focus on complex controllers such as robust controller, MPC, or sliding mode controllers, which increase computational complexity and implementation cost^2,28,46^. However, the potential of simple and industry-friendly PID controllers optimized using advanced hybrid algorithms under predicted wind speed disturbances remains less explored.
5. Although several optimization algorithms have been applied individually for controller tuning, hybrid optimization approaches such as PSO–GWO for tuning PID parameters in MG LFC systems have not been extensively investigated^40,42^.
6. Due to the intermittent nature of wind energy, maintaining frequency stability in MGs remains major challenge, there is still a research gap in understanding how improved wind speed prediction methods influence frequency regulation performance in MG-based LFC systems, highlighting the need for robust controller tuning strategies integrated with accurate wind prediction models^25^^[,26^^29,33^.
7. Most existing LFC studies are validated only through simulation results. Limited research has investigated the real-time hardware validation using platforms like OPAL-RT Technologies^29,39,48^. Therefore, experimental verification of the proposed control strategy in a real-time environment remains an important research gap.

### Motivation of the research work contributions

Based on the identified research gaps, the motivation of the research work are as follows,

1. The increasing penetration of wind energy in MGs introduces significant variability and uncertainty in power generation. This motivates the need to integrate accurate wind speed prediction models with LFC to better analyze their impact on system frequency stability.
2. In many practical scenarios, long-term historical wind datasets are not readily available. Therefore, it is important to investigate the effectiveness of prediction models such as DNN and SVR when trained using short-duration datasets.
3. Wind characteristics vary significantly across geographical regions. This motivates the need to evaluate wind prediction models using data from multiple locations to ensure robustness and better generalization of the prediction methods.
4. Most existing LFC studies rely on artificial disturbance signals. Using predicted wind speed and corresponding wind power variations as real disturbance inputs can provide a more realistic assessment of MG frequency dynamics and efficient control to improve the system stability.
5. While many advanced controllers exist, their complexity and high computational requirements limit practical implementation. This motivates the use of a PID controller, which is simple, cost-effective, and widely accepted in industrial applications.
6. To enhance the performance of the PID controller under fluctuating renewable conditions, there is a need to apply hybrid optimization algorithms, such as PSO–GWO, which combine strong exploration and exploitation capabilities for effective parameter tuning in comparison to the conventional PSO and GWO.
7. Although many LFC control strategies are proposed in the literature, their performance is often verified only in simulation environments. This motivates the need to validate the controller performance in real-time using HIL platforms such as OPAL-RT Technologies under wind uncertainties.

### Major contributions of the proposed work

The novelty of this work presents the integration of SVR and DNN-based wind speed prediction models with the LFC of a MG system. The predicted wind speed data from four locations are used as disturbance inputs to a MG consisting of WTG, DEG, FC, BESS, FESS, and UC. A PID controller is employed for frequency regulation, and its parameters are tuned using a hybrid PSO–GWO optimization algorithm. The major contribution of the proposed works is outlined as follows:

1. A comparative study of SVR and DNN models is carried out to predict short-term wind speed using real wind data from four different geographical locations, enabling evaluation of their prediction accuracy and robustness.
2. The proposed study investigates the performance of SVR and DNN models using a short-duration dataset (15 days), demonstrating their capability to perform effective wind speed prediction even when long-term historical data is unavailable.
3. The predicted wind speed and corresponding wind power variations are incorporated as disturbance inputs to a MG system, allowing the investigation of their impact on frequency stability under realistic renewable energy fluctuations.
4. A MG model consisting of WTG, DEG, FC, BESS, FESS, and UC is developed to analyze the load frequency control problem under predicted wind speed disturbances at four different locations.
5. A PID controller is employed for LFC due to its simple structure, reliability, and ease of implementation, and its parameters are optimally tuned using a hybrid PSO–GWO optimization algorithm to enhance frequency regulation performance.
6. The proposed hybrid PSO–GWO optimized PID controller compared with PSO tuned PID and GWO tuned PID to demonstrates improved dynamic performance and enhanced frequency stability of the MG under predicted wind speed disturbances.
7. The stability and convergence characteristics of the proposed control strategy are analyzed using frequency-domain Bode plots and Box-and-Whisker plots. Furthermore, the effectiveness of the proposed method is validated in a real-time hardware-in-the-loop environment using the OPAL-RT, demonstrating its practical applicability.

The remaining subsequent sections of this paper are structured as follows: Sect. 2 presents the detailed modelling of the MG system. Section 3 describes the optimization techniques and controller employed in the study. Section 4 discusses the modelling of the wind speed prediction models. Section 5 presents the results and analysis and finally, the conclusion Sect. 6 summarizes the main findings of the study and outlines future research scopes. The proposed work aligns with the objectives of sustainable development goal (SDG), i.e., SDG 7 i.e., affordable and clean energy and SDG 13 i.e., climate action), as it focuses on enhancing the stability, reliability, and efficient integration of renewable energy sources in microgrids thereby promoting sustainable, clean, and resilient energy systems while supporting efforts to mitigate climate change.

## System modeling

**Fig. 1:**
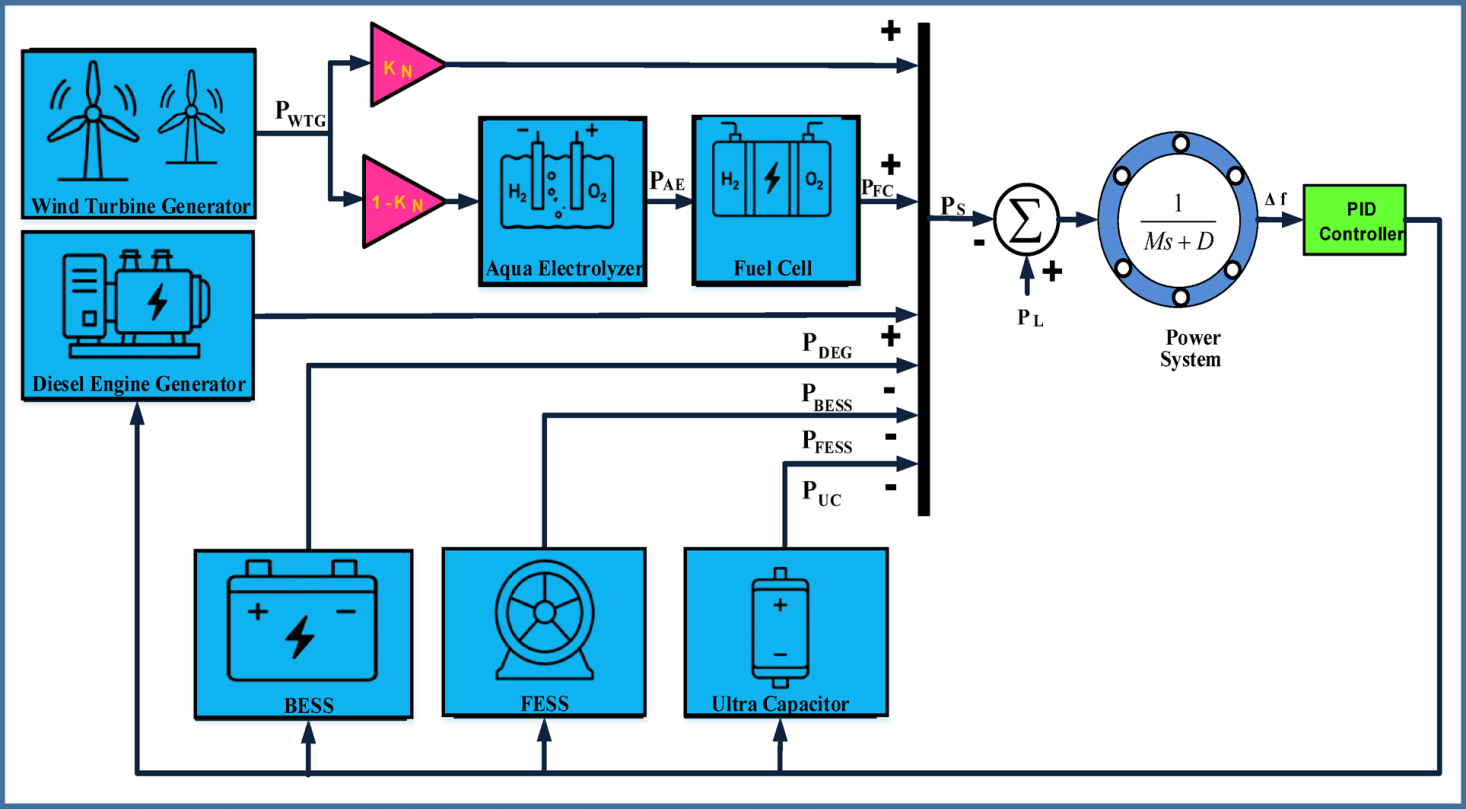
Configuration of the MG system.

**Fig. 2:**
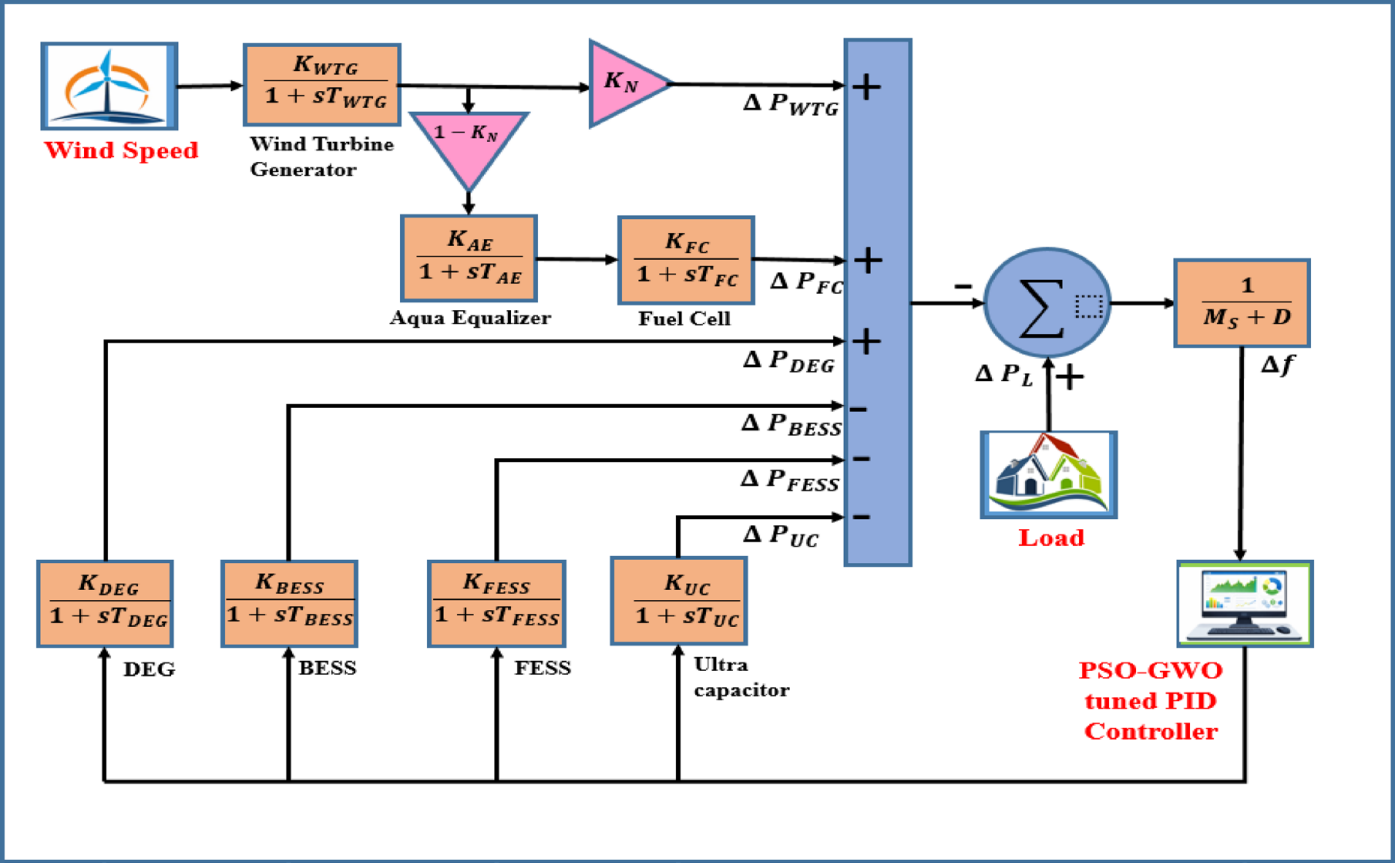
Transfer function Model of MG system.

This section presents the configuration and mathematical modelling of the MG system. The configuration of the MG is illustrated in Fig. 1, while the corresponding transfer function model is shown in Fig. 2. The MG primarily consists of a WTG supported by a DEG to ensure reliable power generation. To further enhance system efficiency, an AE and FC are integrated with the WTG. Moreover, the impact of wind power intermittency is mitigated by incorporating energy storage systems, including a BESS, FESS, and UC, which collectively improve system stability and dynamic performance^11,29,30^. Finally, for testing the robustness of the proposed scheme, appropriate rate constant nonlinearities were added to the storage devices like BESS, DEG, FESS and UC and are modelled as first order transfer function (TF) with saturation block. The following nonlinearities constraints were considered for incorporating electromechanical constraints such as $\left| {{{\dot P}_{BESS}}} \right|$ <0.05,$\left| {{{\dot P}_{FESS}}} \right|$ < 0.02, $\left| {{{\dot P}_{DEG}}} \right|$ < 0.1, $\left| {{{\dot P}_{UC}}} \right|$ < 0.03. The brief introductions and modelling of various components of MG are as follows:

### Wind turbine generator

The WTG considered in this work can be modelled as linear TF neglecting the nonlinearities and can be given as follows^38^;

1 $${G_{WTG}}\left( s \right)=\frac{{{K_{WTG}}}}{{1+s{T_{WTG}}}}$$

Where *K*_*WTG*_ and *T*_*WTG*_ represent the gain and time constants respt. related to WTG. The mechanical power output of the WTG is given by;

2 $${P_w}=\frac{1}{2}\rho {A_r}{C_p}V_{W}^{3}$$

Here, air density, swept area of the blades are symbolised as ρ and *A*_*r*_ respt. and *C*_*p*_ depends on tip speed ratio *λ* and blade pitch angle *β*. The expression for *C*_*p*_ is given by

3 $${C_P}=\left( {0.44 - 0.0167\beta } \right)sin\left[ {\frac{{\pi \left( {\lambda - 3} \right)}}{{15 - 0.3\beta }}} \right] - 0.0184\left( {\lambda - 3} \right)\beta$$

Here, *λ* is expressed as;

4 $$\lambda =\frac{{{R_{blade}}{\omega _{blade}}}}{{{V_W}}}$$

Where,${V_W}$ is the wind speed, *ω*_*blade*_ is the rotational speed of the blades and *R*_*blade*_ is the radius of the blades. The wind turbine output of the wind turbine generator is simultaneously supplied directly to the system and to the fuel cell through an aqua electrolyser.

### Diesel engine generator (DEG)

The DEGs are the autonomous power generation systems which work independently to meet the required power deficit to maintain the power balance. DEG is modelled as linear TF^29^:

5 $${G_{DEG}}\left( s \right)=\frac{{{K_{DEG}}}}{{1+s{T_{DEG}}}}$$

Where ${K_{DEG}}$ and ${T_{DEG}}$ are gain and time constant of DEG.

### Battery energy storage system (BESS)

BESS is the supplementary storage device which has of the capability of controlling of controlling active and reactive powers. The TF of BESS^30^ is represented by Eq. 6.

6 $${G_{BESS}}\left( s \right)=\frac{{{K_{BESS}}}}{{1+s{T_{BESS}}}}$$

Where ${K_{BESS}}$ and ${T_{BESS}}$are the gain and time constants of BESS.

### Flywheel based energy storage system (FESS)

FESS is a kinetic energy storage system that accumulates energy in a flywheel rotor for further utilization. This is a highly versatile energy storage device which offers higher degree of control over the output and decides how much power to be injected. It is shown as a linear transfer function given as^29^;

7 $${G_{FESS}}\left( s \right)=\frac{{{K_{FESS}}}}{{1+s{T_{FESS}}}}$$

Where ${K_{FESS}}$ and ${T_{FESS}}$ represent gains and time constants of FESS.

### Ultra capacitor (UC)

The UCs are one of the efficient energy storage devices which offer larger charge storing capability. It stores energy during power surplus and supplies the power during deficit at shorter interval of time. It has double layer with large surface area and filled with carbon. It provides a much greater value of capacitances as compared to conventional capacitors of same size and can be effectively used to maximize the output and provide stability to the grid^32^. The UC transfer function is:

8 $${G_{UC}}\left( s \right)=\frac{{{K_{UC}}}}{{1+s{T_{UC}}}}$$

Here, ${K_{UC}}$ and ${T_{UC}}$stand for gains as well as time constants of UC.

### Fuel cell (FC) system

The FCs are the devices where hydrogen generated in aqua electrolyser is directly fed into the fuel cell where chemical reaction of electrolysis takes place so that current is generated as electrons flow from anode to cathode and proton flows through the electrolyte from cathode to anode. The TF of FC can be given as;

9 $${G_{FC}}\left( s \right)=\frac{{{K_{FC}}}}{{1+s{T_{FC}}}}$$

Where, *K*_*FC*_ and *T*_*FC*_ are gain and time constants of FC.

### Aqua electrolyzer (AE)

The stochastic energy produced from the wind turbine is partially fed to aqua electrolyser for producing hydrogen that can produce electricity when interacting with oxygen through the process of electrolysis. The TF of AE is;

10 $${G_{AE}}\left( s \right)=\frac{{{K_{AE}}}}{{1+s{T_{AE}}}}$$

Where, *K*_*AE*_ and *T*_*AE*_ are gain and time constants of AE.

### Modelling of power system

The power system considered in the proposed scheme can be modelled as;

11 $$G\left( s \right)=\frac{1}{{sM+D}}$$

*Where*,* M* and *D* represent moment of inertia and damping coefficient respt. of the synchronous generator. The parameters of the MG are presented in Table 1.

**Table 1:** Parameters of the MG system^32^.

Component	Gain	Time Constant
WTG	*K*_*WTG*_ = 1.2	*T*_*WTG*_ =1.3
AE	*K*_*AE*_ =0.004	*T*_*AE*_=0.5
FC	*K*_*FC*_= 0.01	*T*_*FC*_= 4
FESS	*K*_*FESS*_=0.04	*T*_*FESS*_=0.3
BESS	*K*_*BESS*_=0.003	*T*_*BESS*_=0.1
UC	*K*_*UC*_=0.8	*T*_*UC*_=0.7
DEG	*K*_*DEG*_=0.002	*T*_*DEG*_=4

## Wind speed prediction

**Fig. 3:**
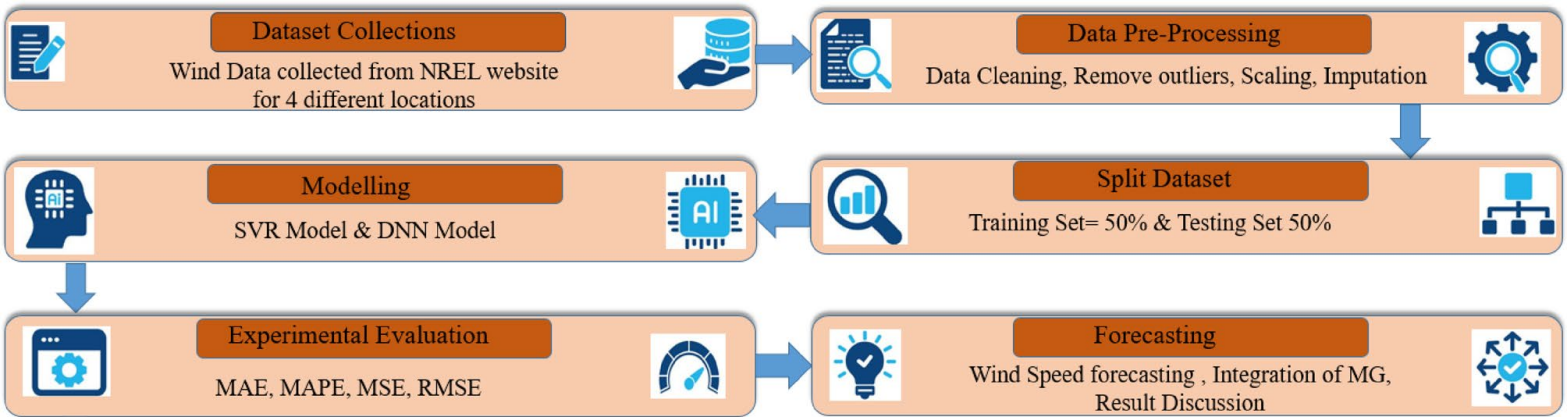
Workflow of Proposed Wind Forecasting Methods: SVR and DNN.

In this section, two different methods namely support vector regression (SVR) and deep neural network (DNN) are described along with their mathematical background for prediction of wind speed and power^38^.The forecasting flow chart is shown in Fig. 3.

### Support vector regression (SVR)

**Fig. 4:**
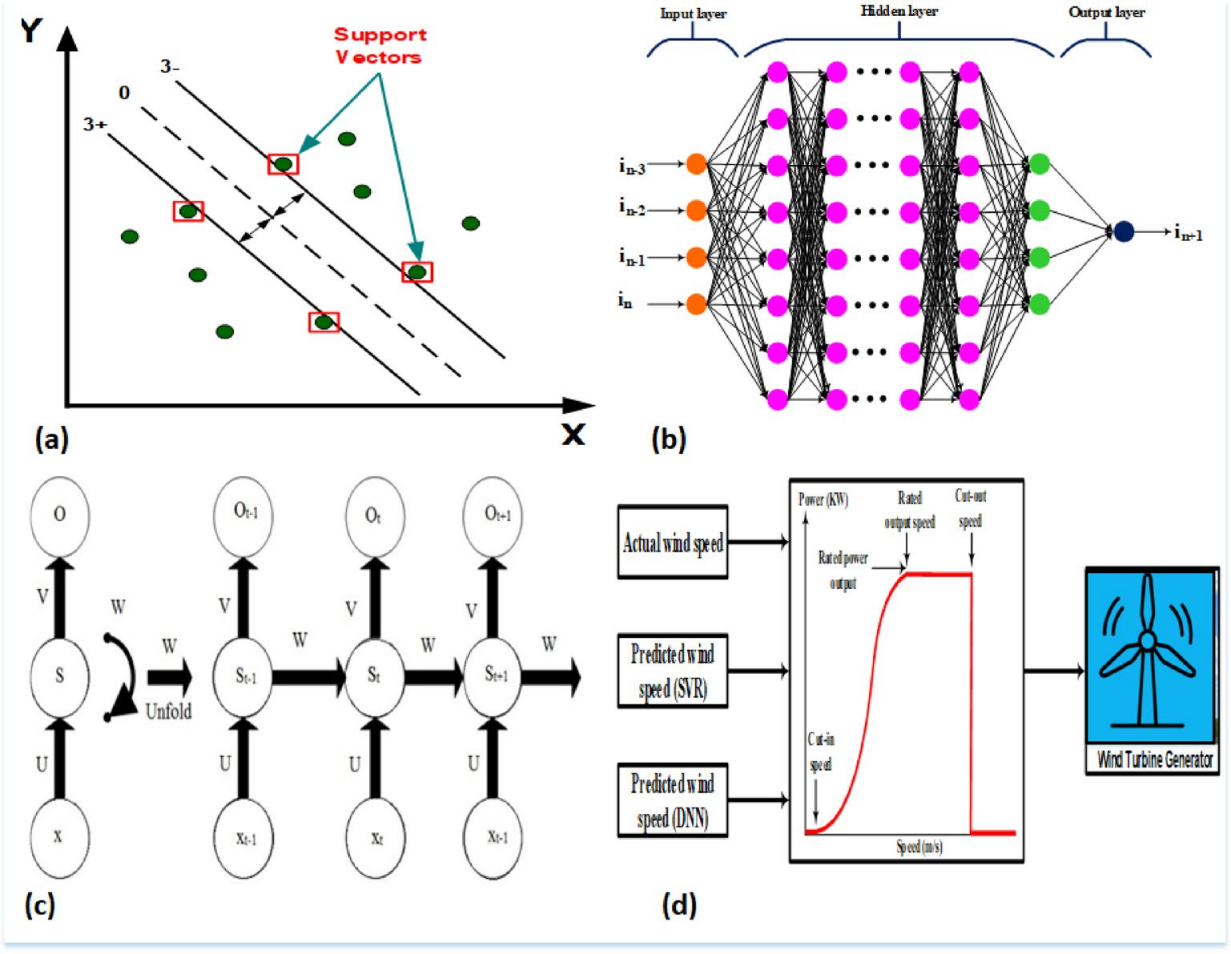
(**a**) Optimal hyper plane of SVM. (**b**) Generalized structure of DNN. (**c**) Working of Recurrent Neural Network. (**d**) Proposed control structure.

The SVR is a machine learning model introduced by Vapnik et al^49^. It is trained by convex optimization technique. The unique feature of SVR is the kernel trick by which the data can be transferred to a non-linear and high dimensional space. It generates sparse prediction function by choosing selected points from the training set which act as support vectors. The advantage of SVR is its understanding of structural risk minimization. While Support Vector Machines are also used for classification, but there exist differences in its operation as a regressor. A continuous function that adapts and best fits the data is used in regression which makes it valuable for wind speed prediction. The optimal hyper plane of SVM is as in Fig. 4a.

The wind speed data is expressed as ${\left\{ {{x_i},{y_i}} \right\}^n}~i=1$, where ${y_i} \in R$ is the time series of wind speed and x_i_ is the historical data. The output of SVR is formulated as [51]:

12 $$f\left( x \right)=w\varphi \left( {{x_i}} \right)+b$$

Where $\varphi :{R^3} \in {R^S}$ is a non-linear map that transforms the wind data collected at the four sites to higher dimension with the help of support vectors. Linear regression is then used to train the SVR in the high dimensional space and predict the future wind speed. Here, $w \in {R^s}$ is the weight vector and $b \in R~$ is the bias. A *ε*-insensitive loss function finds the error between actual and predicted value^16^:

13 $$\left| {y - f\left( x \right)} \right|=\left\{ {\begin{array}{*{20}{c}} 0&{\left| {y - f\left( x \right)} \right| \leqslant \varepsilon } \\ {\left| {y - f\left( x \right)} \right| \leqslant \varepsilon }&{\left| {y - f\left( x \right)} \right| \geqslant \varepsilon } \end{array}} \right.$$

The optimal problem can be described as:

14 $$\begin{gathered} \hbox{min} \frac{1}{2}{\left\| w \right\|^2} \hfill \\ s.t.\;\;{y_i} - w.\phi \left( {{x_i}} \right) - b \leqslant \varepsilon \hfill \\ w.\phi \left( {{x_i}} \right)+b - {y_i} \leqslant \varepsilon \quad \quad i=1,....,n \hfill \\ \end{gathered}$$

$\xi _{i}^{*}$ and ${\xi _i}$ measure the training wind speed outside the ε sensitive zone. The optimal problem is formulated as:

15 $$\begin{gathered} \hbox{min} \frac{1}{2}{\left\| w \right\|^2}+C\sum\limits_{{i=1}}^{n} {\left( {{\xi _i}+\xi _{i}^{*}} \right)} \hfill \\ s.t.\;\;{y_i} - w.\phi \left( {{x_i}} \right) - b \leqslant \varepsilon +{\xi _i} \hfill \\ w.\phi \left( {{x_i}} \right)+b - {y_i} \leqslant \varepsilon +\xi _{i}^{*}\quad \quad \hfill \\ \end{gathered}$$

Here, *C* is the penalty parameter that determines the amount of slack used and calculates the balance between the empirical error and model complexity.

### Deep neural network (DNN)

The DNN is based on replicating how neurons in a brain work. DNN architecture is based on multiple hidden layers with large number of neurons/layers. The structure of DNN with multiple hidden layers is shown in Fig. 4a. It has proven to be successful in understanding layers of information with high level of abstraction present. DNN methods have given ground-breaking results in image and video processing. The best aspect of deep neural network compared to supervised learning is its inherent ability to extract features from abstract data^27^.

Here in the current prediction study, a special type of deep neural network algorithm called recurrent neural network (RNN) is used which is as shown in Fig. 4c. Such architecture has already proven to be capable of predicting sequence of text from future values. It is basically a deep neural net operating with back propagation. Artificial neurons receive data from previous time step and use it to map the future values^32^. Before the evolution of deep neural network, one of the biggest challenges in implementing recurrent neural network was if it had too many time steps then the weights became too large or too small. This is referred to as vanishing gradient problem. To solve this problem, a new model called long short-term memory (LSTM) was developed^5^. In this method, the hidden layer is replaced by complex structure consisting of gates which trap the error.

The mathematical symbols denoted in the Fig. 4b are *x*_*t*_ which stands for the input vector during time *t*, ${s_t}$ represents the hidden state at time *t*, computed from the input vector and the preceding hidden state. It is defined by the equation ${s_t}=f\left( {U{x_t}+W{S_{t - 1}}} \right)$, where *f* signifies the activation function, *U* denotes the weights of the hidden layer, and W represents the transition weights of the hidden state k. The output at time $t~$ can be articulated based on the aforementioned rationale as follows:

16 $${O_t}=f\left( {V{s_t}} \right)$$

Here *V* is the weight of the output layer. Although, RNN models time series accurately, but suffers from vanishing gradient thus LSTM is used to combat it by adding memory composed of input, output, forget gates along with a self-recurrent neuron. The input and output gates regulate the state of the memory cells, while the forget gate determines whether to retain or discard the current state of the memory cells.

## Optimization techniques

The robustness of proposed GWO-PSO, PSO and GWO techniques are tested by calculating the ISE of the frequency variation $\Delta f$in MG. ISE is considered in the objective function (J) which is used in the optimization process to minimize the deviation in the system frequency^32,38^:

17 $$J=Objective~function=ISE=\smallint \Delta {f^2}\left( t \right)dt$$

### Particle swarm optimization (PSO)

PSO is modelled based on the behaviour of a group of birds moving from one place to other where each particle is allowed fly in search space^34^ with a specific position and velocity with respect to that of the whole group:

18 $$v_{i}^{{k+1}}=\omega v_{i}^{k}+{c_1}ran{d_1}\left( {P_{{i,pbest}}^{k} - x_{i}^{k}} \right)+{c_2}ran{d_2}\left( {P_{{i,gbest}}^{k} - x_{i}^{k}} \right)$$

*ω* is the initial weight=(0.4,0.9), random variables *rand*_1_ and *rand*_2_ that vary in (0,1) and *c*_*1*_and *c*_*2*_ are acceleration coefficients. The position of each swarm is updated using:

19 $$x_{i}^{{new}}={x_i}+{v_i}$$

20 $$x_{i}^{{k+1}}=\left\{ {\frac{{{x_{i,ne{w^{iff\left( {{x_{i,new}}} \right) \leqslant f\left( {{x_i}} \right)}}}}}}{{x_{i}^{{otherwise}}}}} \right\}$$

### Grey wolf optimization (GWO)

Metaheuristic algorithm GWO uses the intelligence of a group of Grey-Wolves (GWs) pursuing prey. GWs folow an intelligent technique by managing their own position with respect to the group to capture the prey efficiently. This process is modelled mathematically, where a fittest solution has been assigned to the *α* group of GWs followed by $\left( {\beta ,\gamma \& \delta } \right)$ groups. The GWs try to formulate a boundary around the prey and then employ the hunting process to reach the victim:

21 $$\vec {D}=\left| {\vec {C}{{\vec {X}}_P}\left( t \right) - \vec {X}\left( t \right)} \right|$$

22 $$\vec {X}\left( {t+1} \right)=\left| {{{\vec {X}}_P}\left( t \right) - \vec {A}\vec {D}} \right|$$

*X* and *Xp* are vectors representing the position of GWs and prey respectively; *A* and *C* are vector coefficients:

23 $$\vec {A}=2.\vec {a}\overrightarrow {{r_1}} - \vec {a}$$

24 $$\vec {C}=2\overrightarrow {{r_2}}$$

*r*_*1*_ and *r*_*2*_ are random vectors varying in [0, 1] and the parameter ‘*a*’ starts with 2 and decreased slowly to 0. The mathematical model is presented as below:

25 $$\overrightarrow {{D_\alpha }} =\overrightarrow {{C_1}} .\overrightarrow {{X_\alpha }} - \vec {X},\overrightarrow {{D_\beta }} =\overrightarrow {{C_2}} .\overrightarrow {{X_\beta }} - \vec {X},\overrightarrow {{D_\delta }} =\overrightarrow {{C_3}} .\overrightarrow {{X_\delta }} - \vec {X}$$

26 $$\overrightarrow {{X_1}} =\overrightarrow {{X_\alpha }} - \overrightarrow {{A_1}} \left( {\overrightarrow {{D_\alpha }} } \right),\overrightarrow {{X_2}} =\overrightarrow {{X_\beta }} - \overrightarrow {{A_2}} \left( {\overrightarrow {{D_\beta }} } \right),\overrightarrow {{X_3}} =\overrightarrow {{X_\delta }} - \overrightarrow {{A_3}} \left( {\overrightarrow {{D_\delta }} } \right)$$

The average of the GW’s position in *α*,* β* and *δ* groups is evaluated using the following equation:

27 $$\vec {X}\left( {t\,+\,1} \right)=\frac{{\overrightarrow {{X_1}} +\overrightarrow {{X_2}} +\overrightarrow {{X_3}} }}{3}$$

### Hybrid PSO-GWO technique

**Fig. 5:**
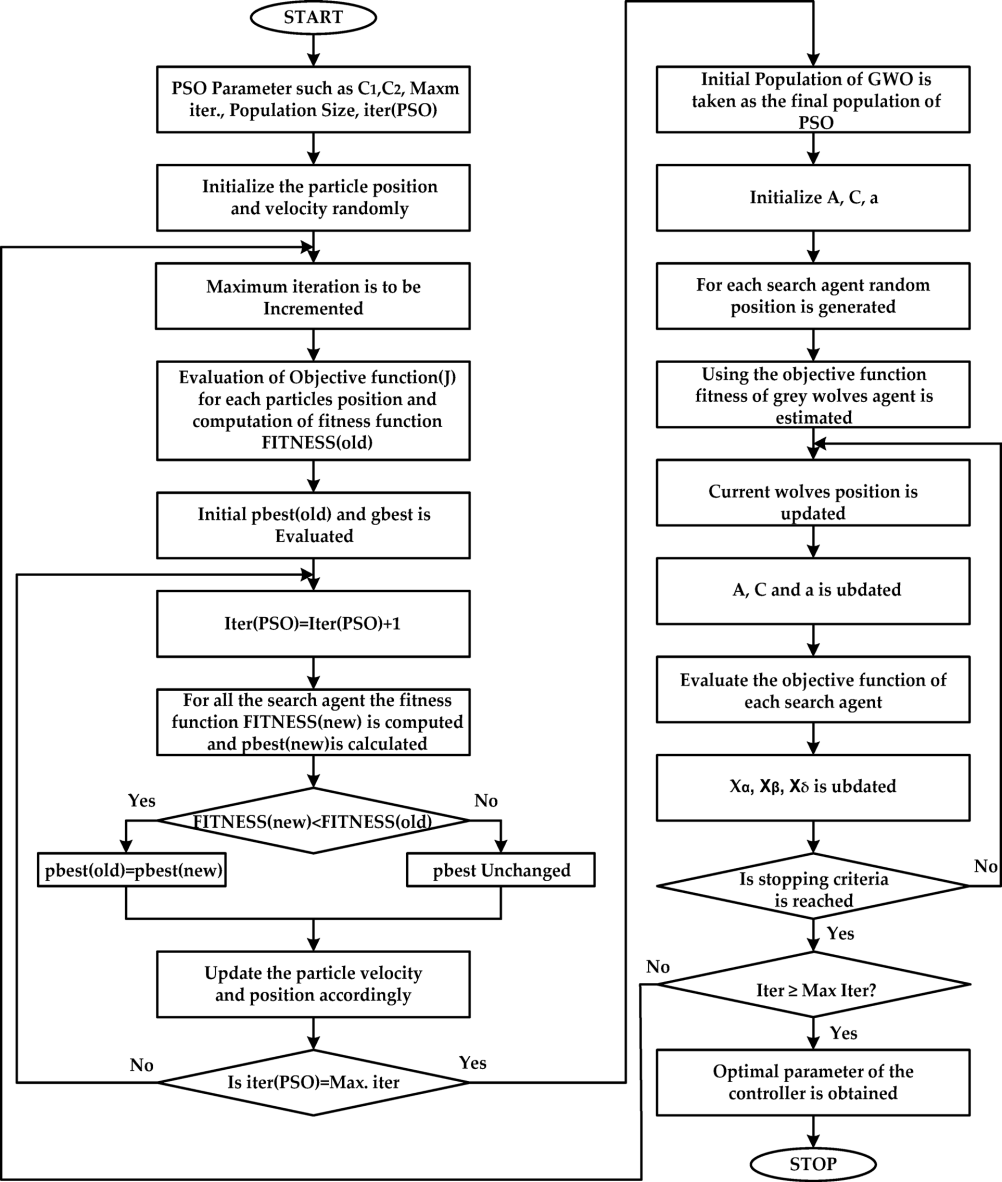
Flowchart for implementing hybrid PSO-GWO.

The individual limitations of PSO and GWO can be mitigated by combining them into a hybrid optimization approach. PSO may suffer from premature convergence and trapping in local optima in complex search spaces, although it is simple to implement and has good convergence characteristics. In contrast, GWO may exhibit slower convergence and limited exploration, but it has strong capability to track the global optimum. Therefore, the hybrid PSO–GWO technique effectively exploits the strengths of both methods, improving optimization performance and frequency regulation in the MG^43^. Therefore, keeping in mind, the merits and demerits of PSO and GWO, a hybrid PSO-GWO optimized PID controller is proposed in this paper to compensate the demerits with the exploitation of the individual merits. In the proposed hybrid approach, PSO performs the primary optimization process, while GWO is periodically invoked within each iteration to refine the global best solution. This co-operative sequential interaction enables the algorithm to maintain a balanced exploration–exploitation trade-off during the optimization process. As a result, the hybrid PSO–GWO technique enhances the controller’s ability to regulate MG frequency effectively by mitigating uncertainties associated with wind power generation^44^. Table 2 presents the parameters of the hybrid PSO–GWO algorithm used during the simulation, while the flowchart illustrating the optimization procedure is shown in Fig. 5. Initially, the PSO algorithm is executed by defining parameters such as C1, C2, maximum iterations, and population size. The particle positions and velocities are randomly initialized, and the PSO process is carried out to perform global exploration of the search space. Subsequently, the GWO algorithm is applied in a sequential manner to enhance exploitation and improve convergence characteristics.

**Table 2:** PSO-GWO parameters used in LFC simulation^43^.

PSO parameters		GWO parameters	
Parameters	Value	Parameters	Value
Population Size	30	Population Size	30
Inertia weight	0.7	Max epochs	50
Cognitive weight	1.5	Alpha weight	1.0
Social coefficient	1.5	Beta weight	1.0
Max iterations	50	Delta weight	1.0

## Results and analysis

Here, simulated results with descriptions are presented in two different sub-sections. First sub-section describes the wind speed prediction results and analysis using the support vector regression and deep neural network. Here, wind speed is predicted using SVR and DNN for the actual wind speed data available at four different sites. Second sub-section presents the impact of wind speed prediction on the frequency regulation in the DG based MG system. The simulations for wind speed prediction and its impact on frequency control in the MG is performed using MATLAB software.

### Wind speed prediction using SVR and DNN

**Fig. 6:**
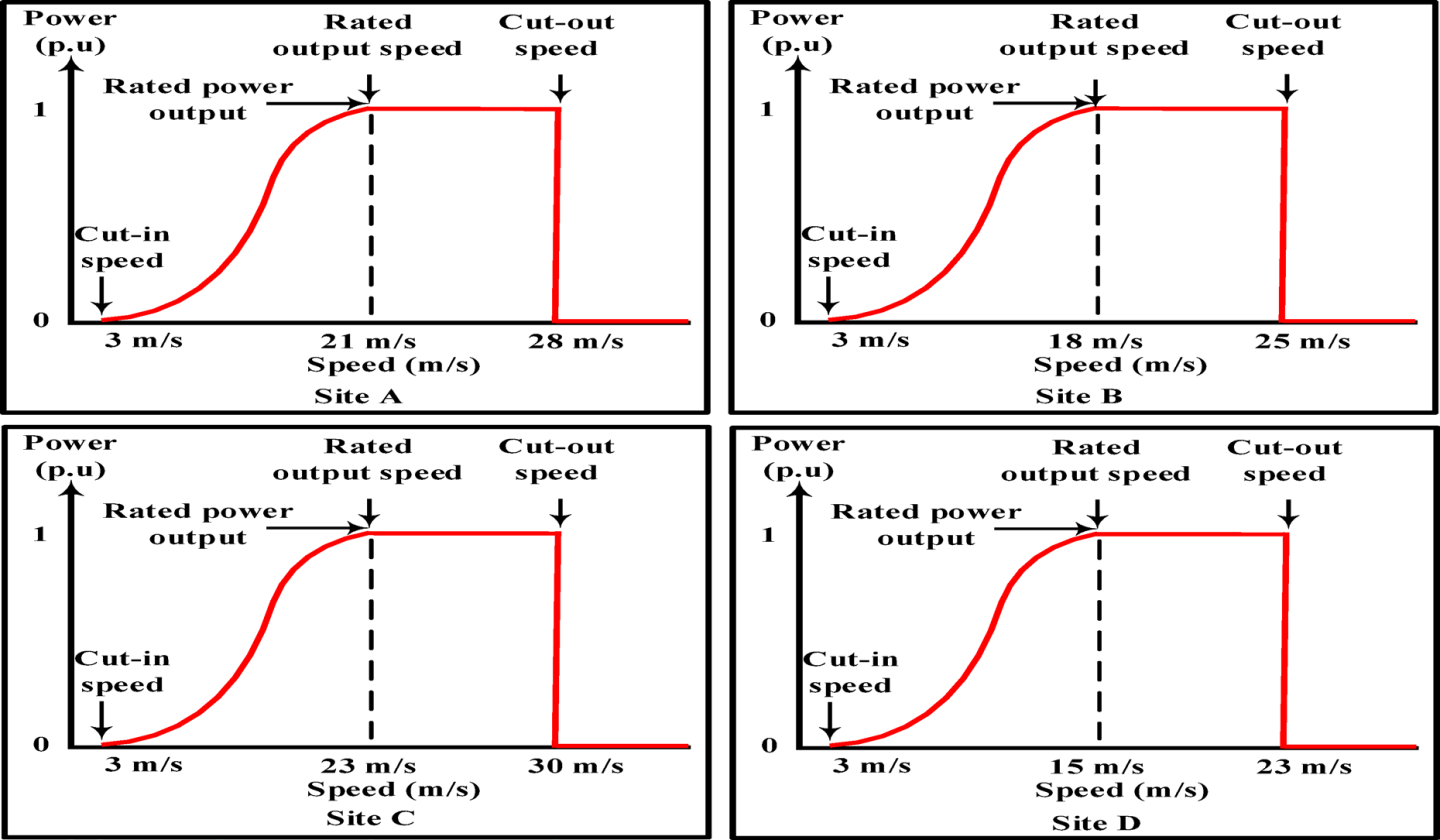
Wind power curve of four different sites.

In this paper, real time wind speed data from four different sites spread across USA, available in National Renewable Energy Laboratory (NREL) website, is considered. The data is taken from both eastern and western dataset of WIND Toolbox. From the considered dataset, Site A is located at longitude of −120.73^0^ and latitude of 35.16^0^. Site B is located at longitude of −98.60^0^ and latitude of 42.36^0^. Site C is located at longitude of 75.23^0^ and latitude of 37.40^0^. Site D is located at longitude of −81.30^0^ and latitude of 25.32^0^. The data is recorded in every five minutes. As the wind speeds vary according to the topography, pressure, air temperature, and density are recorded to encompass different aspects of wind speed at different sites. The wind speed data over a period of one year is observed and the variation over each month is analysed. Figure 4d represents the conversion of wind speed to mechanical power input of the turbine. The wind power curve, as shown in Fig. 6, gives the mechanical power inputs for corresponding wind turbine. The power curve is distinct for each location and is influenced through the historical wind speed of the area and the specifications of the generator. The curves for different sites are also illustrated in the same figure.

The wind power curve gives the correlation of wind speed to power. The wind curves are used during simulation to generate power for corresponding wind speed. Then, data points over a period of 15 days are taken as training data set and that is used for training SVR and DNN. The trained support vector machine and deep neural network is then used for predicting the wind speed for next three days and actual wind speed is observed.

**Fig. 7:**
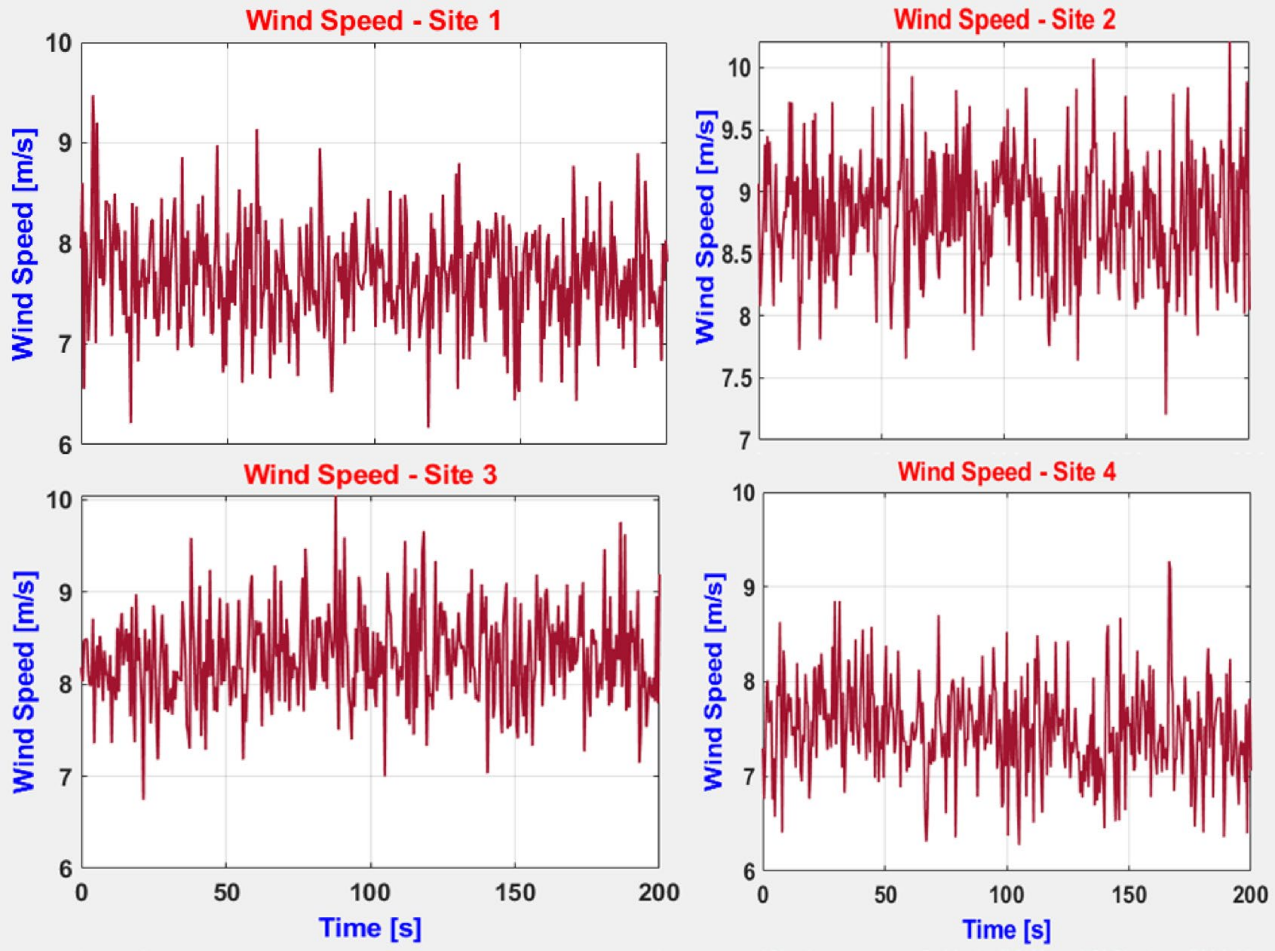
Wind pattern in four sites.

The wind speeds for four different sites are shown in Fig. 7. All sites are located at considerable distance from each other with different topography. Due to such difference in weather conditions and geographical location, the wind speed for each site is distinct in characteristics. The annual wind speed for Site A is 4.83 m/s, for Site B is 8.25 m/s, for Site C is 8.27 m/s and for Site D is 6.09 m/s respectively. An interesting point to notice here is for Site A, the number of samples in the range of speed of 0–5 m/s is particular high due to which the annual wind speed of that particular site is within the range of 5 m/s, whereas the wind speed distribution for other sites is fairly distributed with many samples within the range of 5 to 10 m/s, Consequently, the annual wind speeds at alternative locations exceed 5 m/s.

**Fig. 8:**
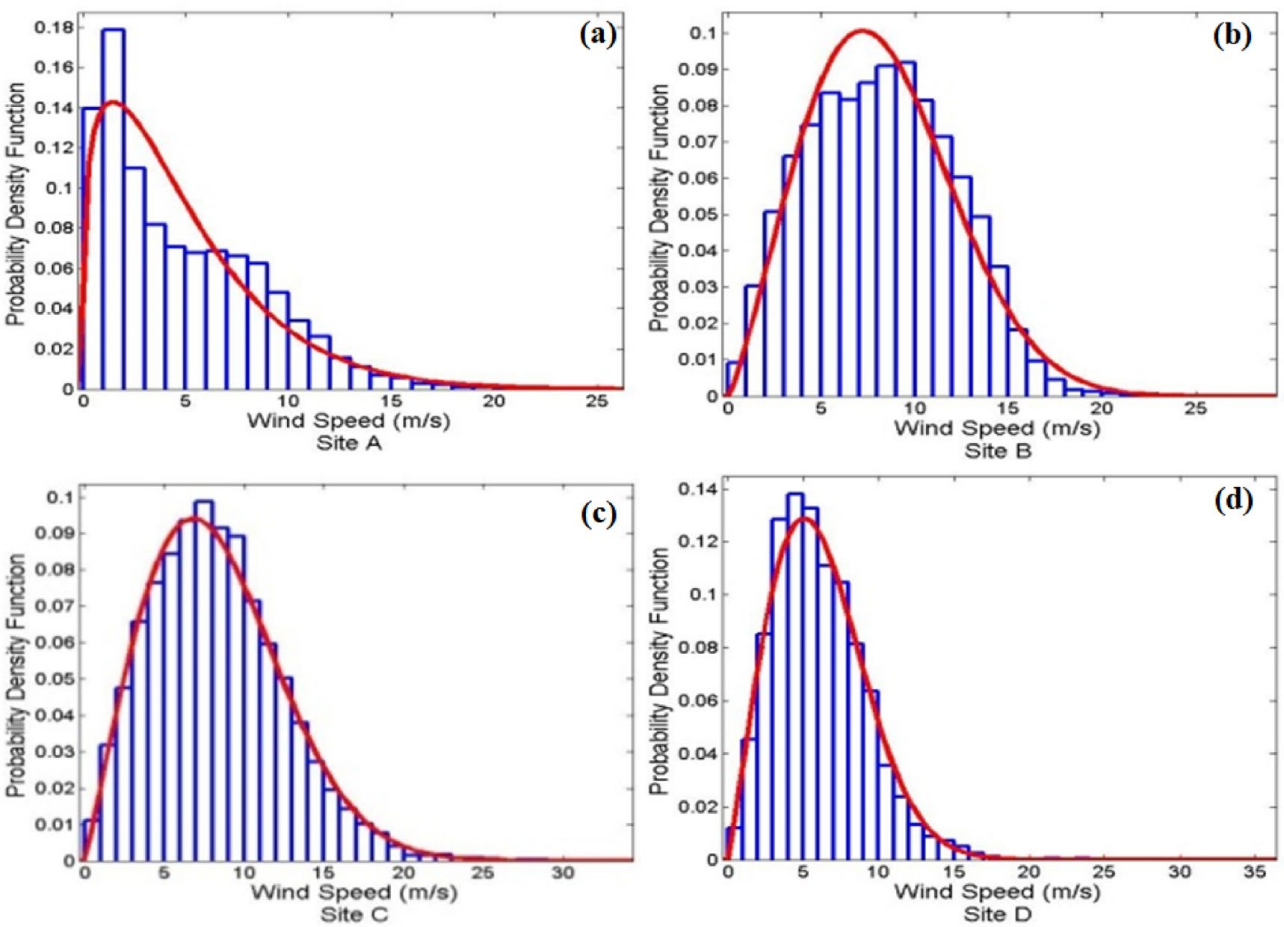
Weibull distribution of wind speed plot of four sites.

The Weibull probability density distribution for wind speed at four different locations, namely Site A, B, C, and D, is illustrated in Fig. 8. It is observed that the Weibull distribution closely fits the annual wind speed data over a one year period. The Weibull distribution curve is commonly recognized as a basic statistical model for wind speed. It is a unimodal continuous distribution including two variables. The probability density function is defined as:

28 $$f\left( {x,\lambda ,k} \right)=\left\{ {\begin{array}{*{20}{c}} {\frac{k}{\lambda }{{\left( {\frac{x}{\lambda }} \right)}^{k - 1}}{e^{ - \frac{x}{{{\lambda ^k}}}}}}&{x \geqslant 0} \\ 0&{x<0} \end{array}} \right.$$

Let *x* represent the variable for which the distribution is to be ascertained, with λ > 0 as the scale parameter and k > 0 as the shape parameter that defines the distribution’s form.

**Fig. 9:**
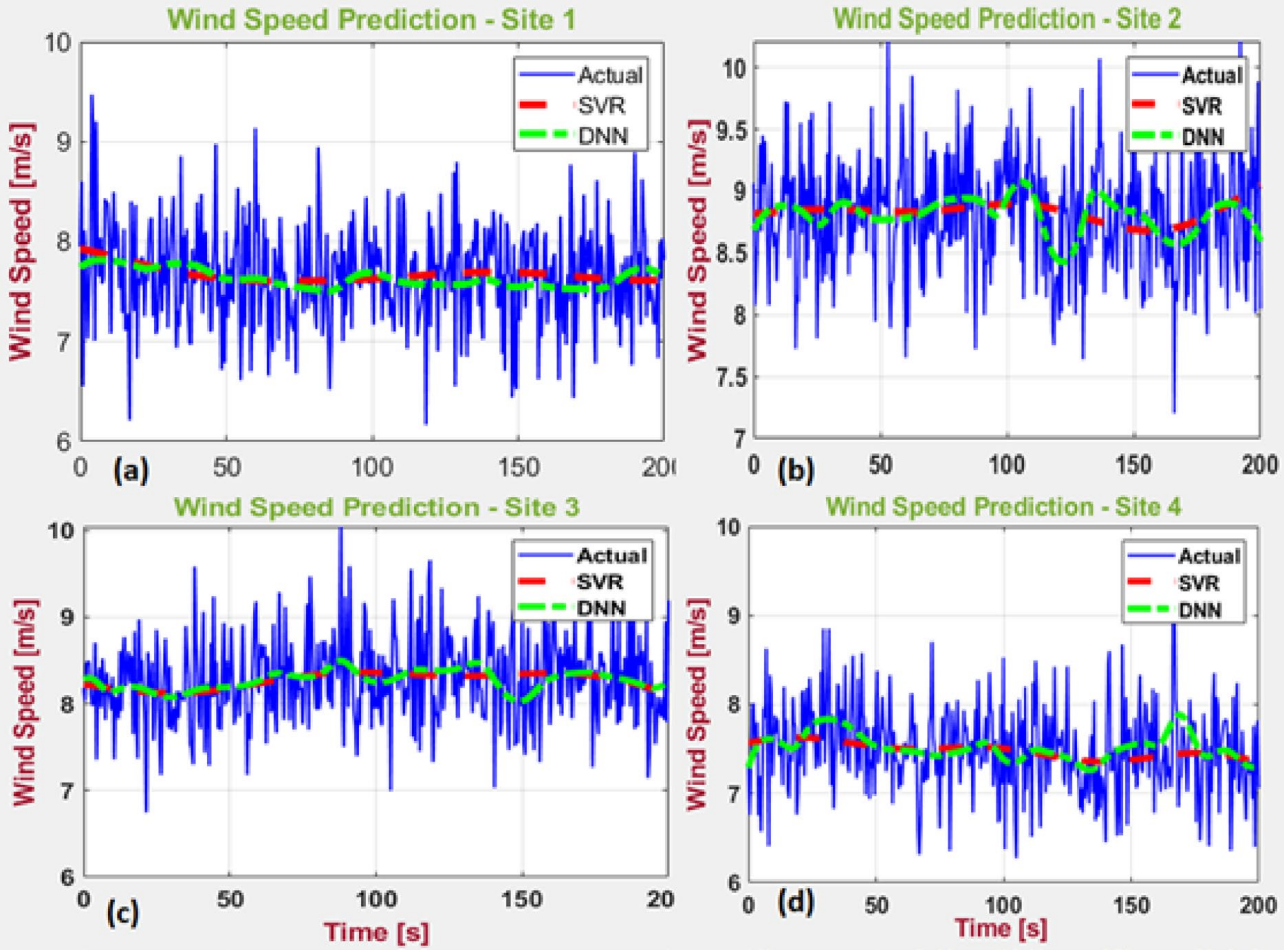
Comparison of actual and predicted wind speeds.

**Fig. 10:**
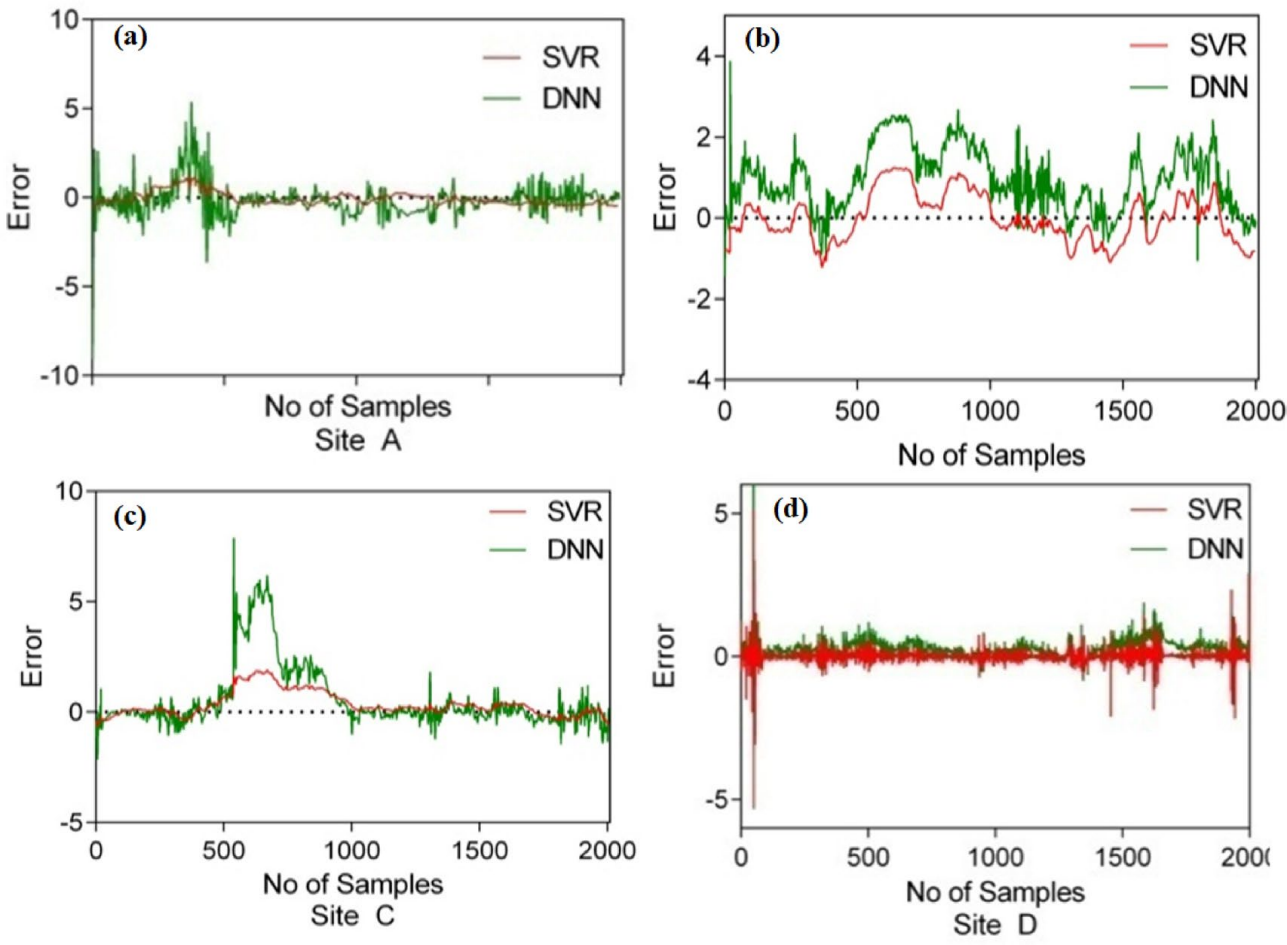
Comparison of error between predicted and actual wind speeds.

The comparison between the actual wind speed and the predicted wind speed using SVR and DNN models is illustrated in Fig. 9. In this study, SVR with a polynomial kernel is implemented using the LIBSVM library^21^, while the DNN model is developed using the Keras library with the Tensor Flow backend^24^. For training the model, 15 days data with each sample collected at an interval 5 min is used, and for validation, another 2000 samples are taken. Again, a comparative analysis between the mean absolute errors of the actual, predicted wind speeds by SVR and DNN is shown in Fig. 10. This also proves than SVR provides minimum error in comparison to DNN at all the four sites.

**Fig. 11:**
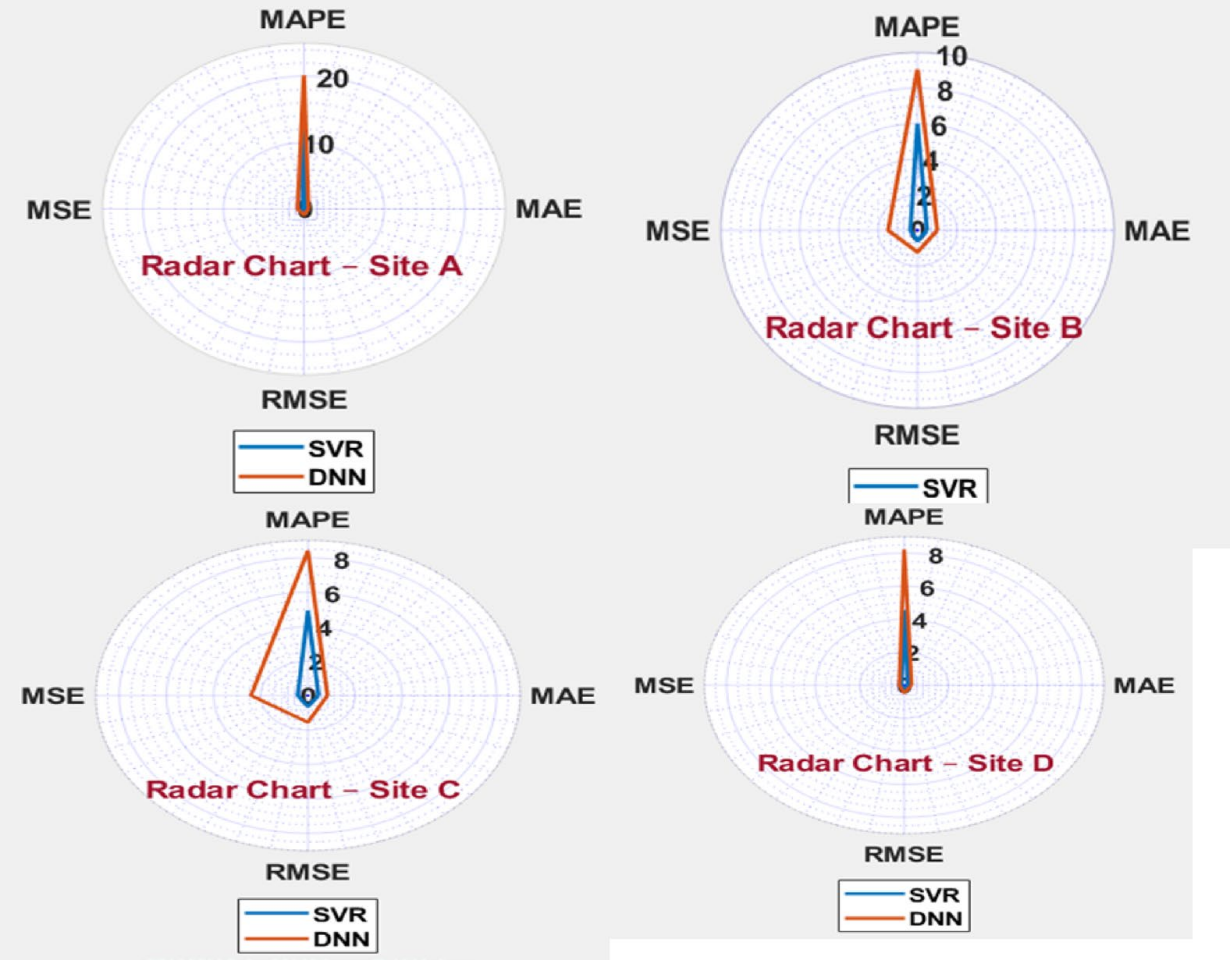
Radar Chart of 4 sites comparing SVR and DNN based outcome.

In addition, a comparative analysis between DNN and SVR for four different sites is presented in Table 3. It can be observed that the MAE, MAPE, MSE, RMSE, confidence intervals, and standard deviations are consistently lower for SVR compared to DNN across all sites (A, B, C, and D). These results clearly indicate that SVR provides more accurate wind speed predictions than DNN. Specifically, SVR achieves reductions of up to 52.94% in MAE, 44.59% in MAPE, 83.54% in MSE, and 59.55% in RMSE across the considered locations, demonstrating its superior forecasting accuracy and robustness. This performance advantage can be attributed to the limited availability of training data, as DNN models typically require large datasets for effective training, whereas SVR performs well even with relatively smaller datasets. Furthermore, the same result are illustrated using radar charts in Fig. 11, which compare SVR and DNN across the four sites based on error metrics (MAE, MAPE, MSE, and RMSE). In these charts, a smaller enclosed area indicates better performance. The plots show that SVR consistently exhibits a smaller radar area compared to the DNN model, further confirming its superior accuracy. Additionally, Table 4 presents a comparative evaluation of the proposed prediction model with existing approaches, highlighting the effectiveness of the proposed method.

**Table 3:** Different errors in wind speeds using SVR and DNN.

Error	Site A		Site B		Site C		Site D	
	SVR	DNN	SVR	DNN	SVR	DNN	SVR	DNN
MAE	0.29	0.57	0.49	1.01	0.45	0.83	0.16	0.34
MAPE	14.5	20.1	6.00	9.04	4.93	8.41	4.56	8.23
MSE	0.13	0.79	0.36	1.49	0.43	2.41	0.12	0.22
RMSE	0.36	0.89	0.59	1.22	0.65	1.55	0.34	0.47
Confidence Intervals	0.34	0.78	0.53	1.30	0.71	1.62	0.41	0.53
Standard Deviations	0.71	1.72	1.15	2.54	1.40	3.12	0.84	1.23

**Table 4:** Comparative analysis of proposed prediction model with existing models.

Ref.	Prediction Algorithm	Performance Parameters
^8^	LSTM	RMSE = 13.19, MAE = 9.38
	NAR	RMSE = 8.35, MAE = 3.96
	HHT-NAR	RMSE = 5.23, MAE = 1.47
	Optimization of HHT-NAR	RMSE = 1.99, MAE = 0.82
^22^	Fine Tree	RMSE = 0.44, MAE = 0.34
	Line Regression	RMSE = 0.29, MAE = 0.18
	SVM	RMSE = 0.32, MAE = 0.21
^41^	KNN	RMSE = 0.71, MAE = 0.53
	SVR	RMSE = 0.69, MAE = 0.51
	RF	RMSE = 0.69, MAE = 0.51
^50^	Liner Regression Method	RMSE = 1.88, MAE = 3.56
	SVR	RMSE = 1.74, MAE = 3.05
	Proposed Model (SVR)	RMSE = 0.34, MAE = 0.16

### Impact of wind speed prediction on system frequency

This section presents the impact of wind speed prediction for four different sites A, B, C & D on the frequency regulation in the MG system as shown in Fig. 1. The actual and predicted wind speed using SVR and DNN are given as input to the MG. Since, the wind speed vary non-linearly with time, the wind power output also varies accordingly. As a result of the variation in output wind power, there is a mismatch developed between the total power generation from all the resources in the MG system and the load demand^40^. This leads to a deviation in the system frequency which must be kept within the specified limit of 1% deviation from the nominal frequency of 50 Hz. Therefore, conventional PID, PSO, GWO as well as hybrid PSO-GWO based methods are employed to optimize the PID gains so that the frequency error will be minimum. The optimal gains of the PID controller using the above optimization techniques are given in Table 5. The whole simulation of the MG is performed using MATLAB/Simulink software.

**Fig. 12:**
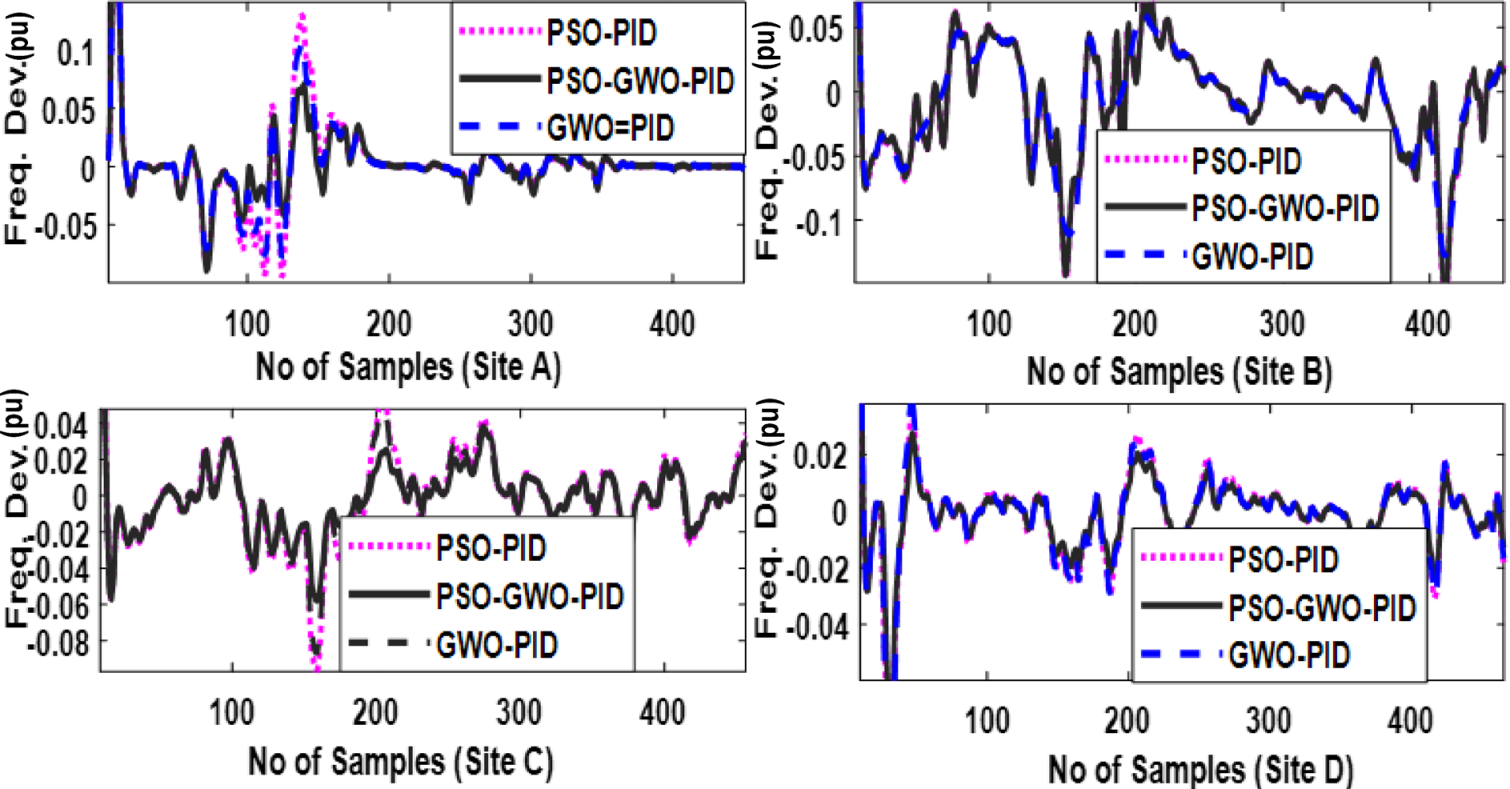
Frequency deviation in the MG due to the predicted wind power using DNN.

The wind speed forecast data for locations A, B, C, and D acts as an input disturbance to the MG system, with the resulting frequency variations depicted in Fig. 12 for PSO-PID, GWO-PID, and hybrid PSO-GWO-PID configurations. Here, the prediction is achieved by DNN and it is seen that deviation in the frequency follows the wind speed input pattern and is minimized by the action of the above controllers for managing the contribution from the resources like FC, DEG and the storage devices. A comparative examination of performance indices (PIs) such as peak overshoot (${M_P}$), settling time (${T_s}$), and ISE is illustrated in Table 5. It is also analysed that the hybrid PSO-GWO optimized PID provides minimum frequency deviation when compared with that of the PSO and GWO optimized PID controllers.

**Fig. 13:**
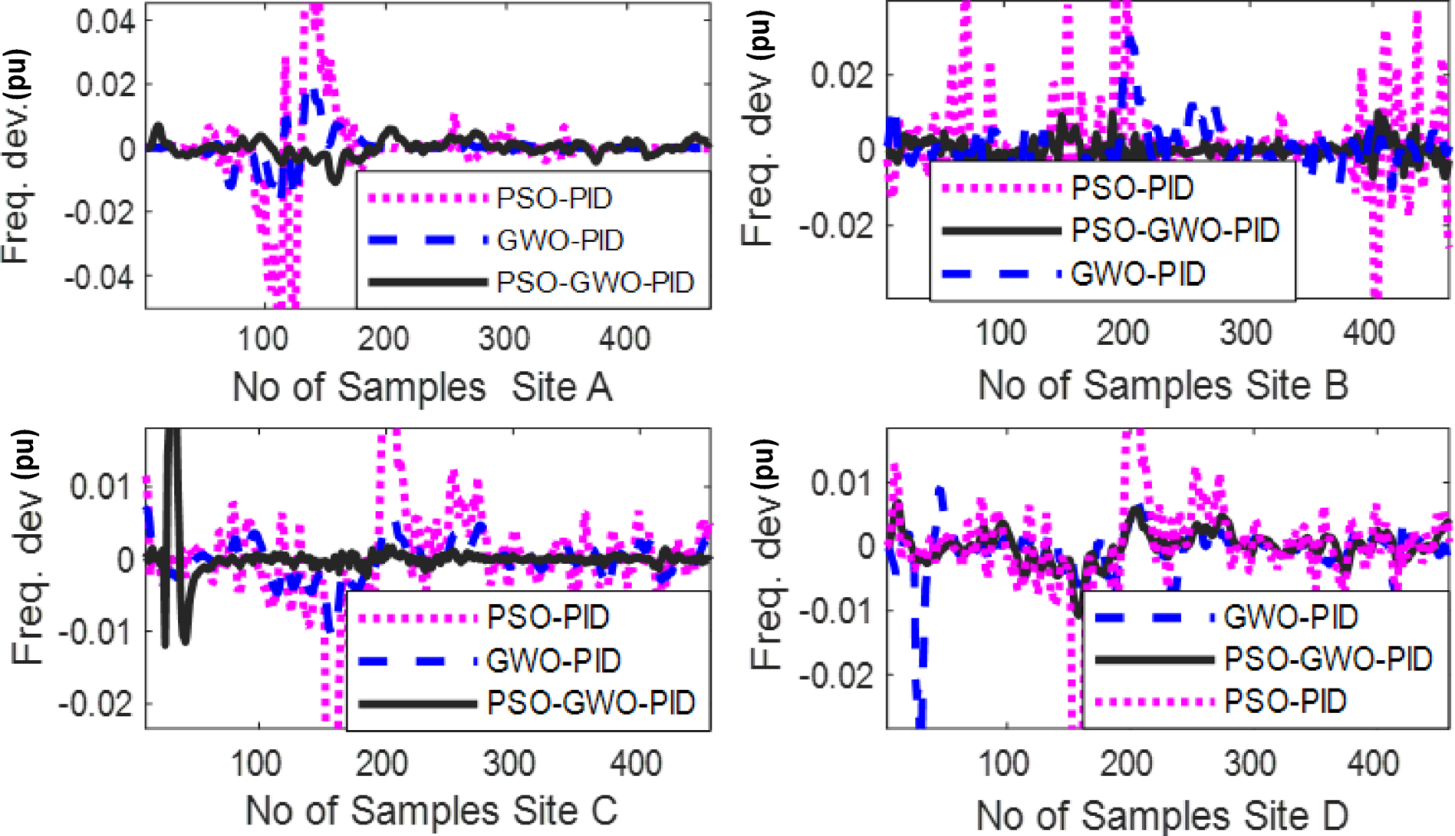
Comparison of frequency deviation in microgrid due to predicted wind power using SVR.

The simulation results for frequency deviation in wind speed prediction utilizing the SVR technique for sites A, B, C, and D are illustrated in Fig. 13 for the PSO-PID, GWO-PID, and hybrid PSO-GWO-PID controllers. The results indicate that the frequency diverges from the designated rated value due to fluctuations in wind speed profiles, yet remains within the prescribed limit through the controller’s intervention. Also, a comparative analysis in terms of the PIs is presented in Table 5. The frequency deviation of the proposed hybrid PSO-GWO optimized PID controller is analysed in comparison to that of the PSO/GWO optimized PID controllers. It is observed that the hybrid PSO–GWO optimized PID controller achieves minimum frequency deviation compared to the PSO- and GWO-optimized PID controllers. The results also indicate that the hybrid PSO–GWO approach consistently outperforms the individual PSO and GWO methods under both wind prediction scenarios across all four sites.

**Fig. 14:**
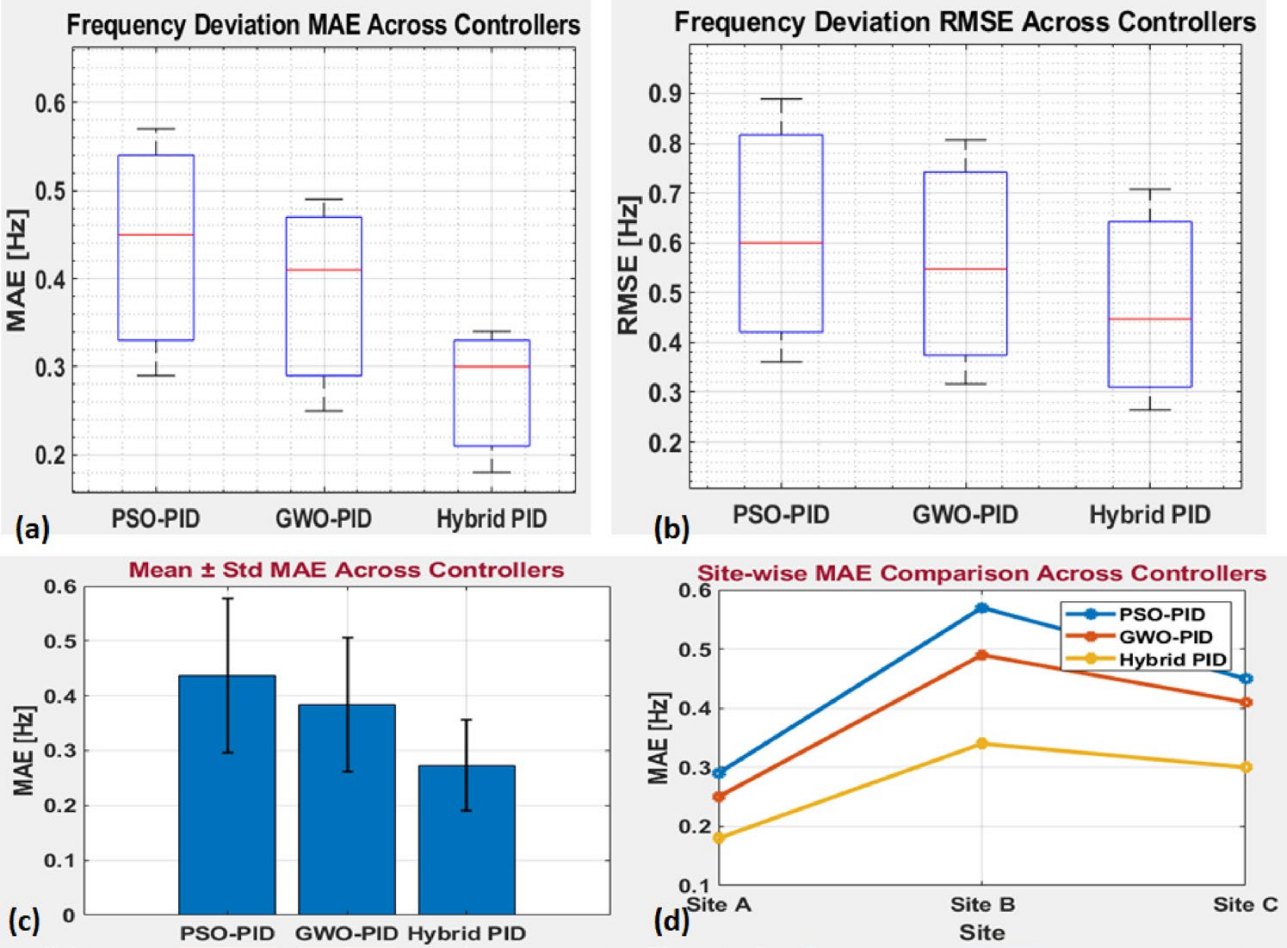
Results comparison with Box and whisker plots.

The comparative performance analysis of PSO-PID, GWO-PID and hybrid PSO–GWO PID controllers using statistical metrics such as MAE and RMSE is presented in Fig. 14. The MAE box plot in Fig. 14a indicates that the hybrid controller achieves the lowest median error along with a significantly reduced spread, reflecting improved accuracy and consistency in minimizing frequency deviations. In contrast, the PSO-PID controller exhibits the highest variability, while the GWO-PID shows moderate improvement. A similar trend is observed in Fig. 14b, where the RMSE analysis demonstrates that the hybrid PSO–GWO PID controller attains the lowest error values with a narrower distribution, confirming its enhanced robustness and superior damping capability. Figure 14c further shows that the hybrid approach yields the lowest mean error and smallest standard deviation, highlighting its reliability compared to the PSO and GWO methods. In addition, the site-wise MAE comparison in Fig. 14d confirms that the hybrid controller consistently outperforms the other controllers across all locations, demonstrating its effectiveness under varying operating conditions.

**Table 5:** Comparison of Gain Values and Performance Indices (PIs) of PSO, GWO and PSO-GWO using SVR and DNN.

Wind Speed Prediction Method	Gains/PIs	Controller Technique								
		PSO			GWO			PSO-GWO		
SVR	PID gains (Kp, Ki, Kd)	0.28	1.73	0.38	0.62	2.27	1.02	0.85	3.82	1.98
	PIs (Mp, Ts, ISE)	0.12	186	0.18	0.09	175	0.08	0.06	156	0.04
DNN	PID gains (Kp, Ki, Kd)	0.87	3.52	1.45	1.12	2.83	0.97	1.58	2.35	0.71
	PIs (Mp, Ts, ISE)	0.04	264	0.21	0.02	225	0.16	0.003	208	0.09

**Fig. 15:**
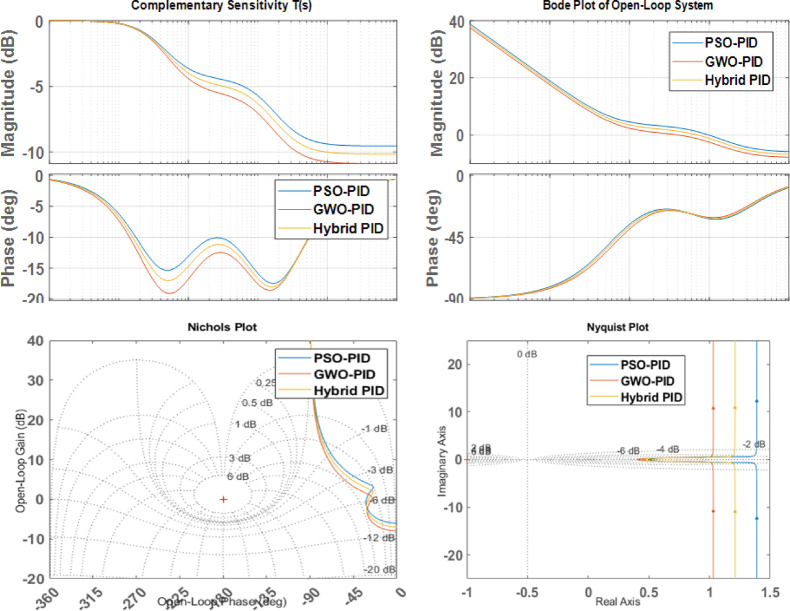
Sensitivity and stability analysis with Bode, Nichols, Nyquist diagrams.

The stability performance of the proposed controllers is further evaluated through frequency-domain analysis using Bode, Nichols, and Nyquist plots, as shown in Fig. 15. In the complementary sensitivity function, the hybrid PSO–GWO PID controller exhibits a lower magnitude at higher frequencies, indicating improved attenuation of noise and disturbances. Additionally, its smoother phase response reflects enhanced stability margins. The Bode plot demonstrates that the hybrid controller maintains a more favorable gain and phase profile, with reduced high-frequency gain, thereby limiting noise amplification and providing improved damping characteristics. The Nichols plot, which integrates gain and phase information for assessing closed-loop robustness, shows that the hybrid controller trajectory remains farther from the critical region around − 180° phase and 0 dB gain, avoiding conditions associated with instability and excessive resonance. Similarly, the Nyquist plot confirms that the hybrid PSO–GWO PID controller maintains a greater distance from the critical point (− 1, 0), indicating an improved stability margin. Overall, the hybrid PSO–GWO PID controller consistently demonstrates superior stability and robustness compared to the PSO- and GWO-based controllers. Additionally, the convergence characteristics of the PSO–GWO PID controller are compared with those of the GWO-PID and PSO-PID controllers for both SVR and DNN cases, based on the results presented in Table 5, as illustrated in Fig. 17. The results indicate that the proposed controller achieves faster convergence and superior performance compared to the other controller methods^47^.

**Fig. 16:**
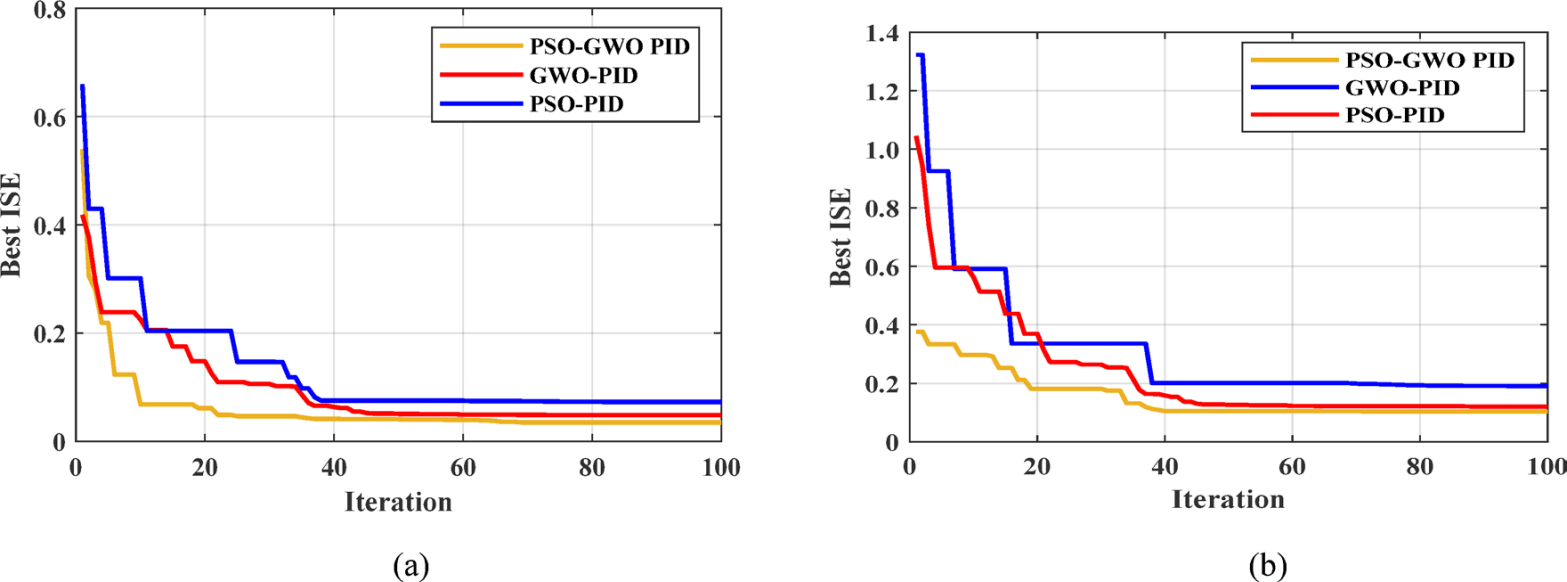
Convergence Curve comparing PSO-GWO PID, GWO-PID, PSO-PID (**a**) SVR (**b**) DNN.

### Real-time stability study using OPAL-RT

**Fig. 17:**
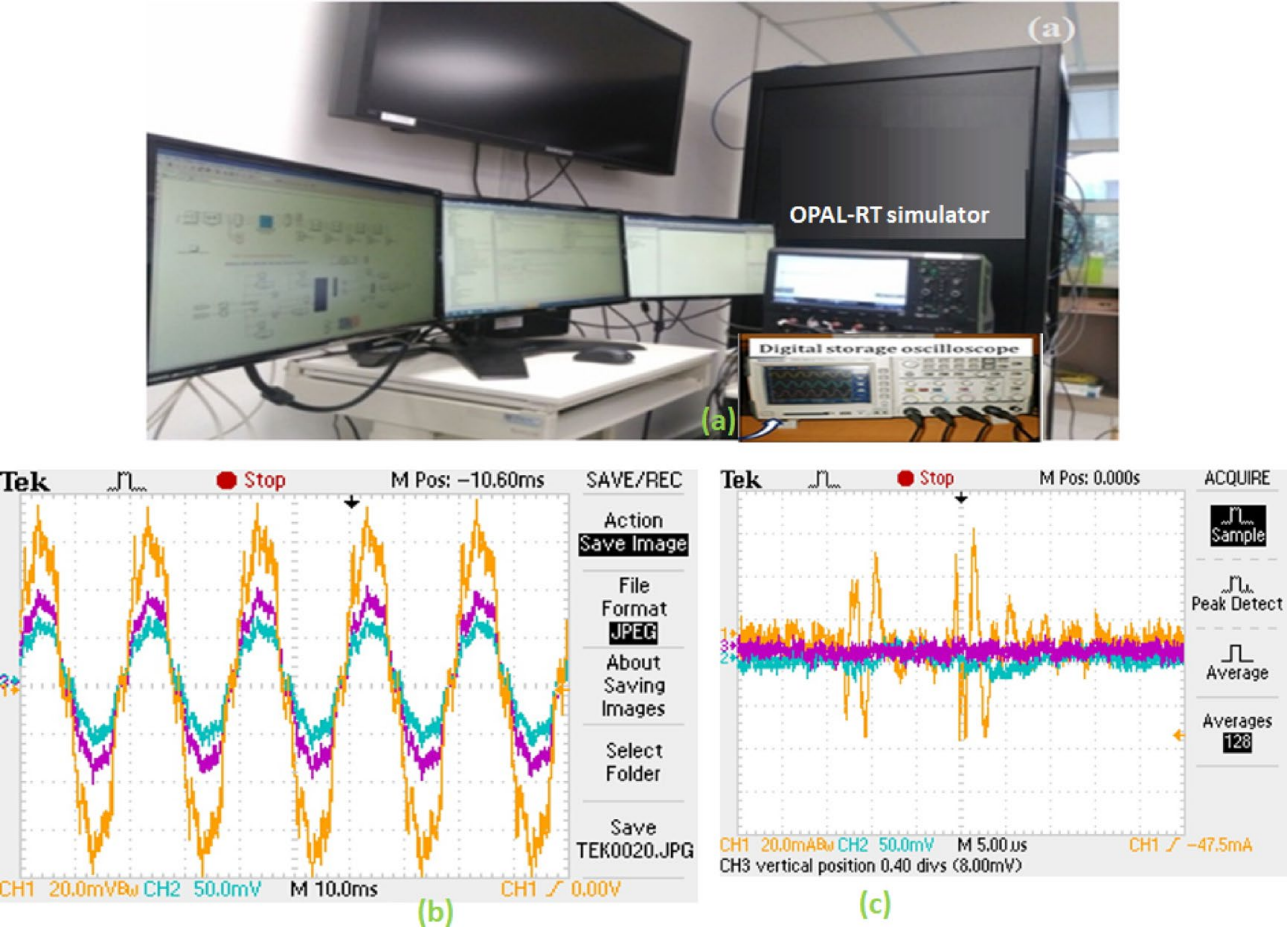
(**a**) Real-time set-up in OPAL-RT (**b**) Deviation in frequency in MG for a step change in wind power (**c**) Deviation in frequency in MG for a continuous step change in wind power (Orange: PSO-PID; Indigo: GWO-PID and Green: hybrid PSO-GWO-PID).

The frequency regulation study using the proposed hybrid PSO-GWO, PSO and GWO optimized PID controllers is tested in this sub-section by using real-time digital simulator OP5600. The e-MEGASIM in OPAL-RT package is used test the stability performance. Figure 17a illustrates the real-time configuration with OPAL-RT. The PC is connected to the OPAL-RT, which operates with FPGA, XILINX v10.1, and MATLAB 2018a. The micro grid model is constructed in RT-LAB, OPAL-RT for real-time validation among wind power uncertainties.

**Fig. 18:**
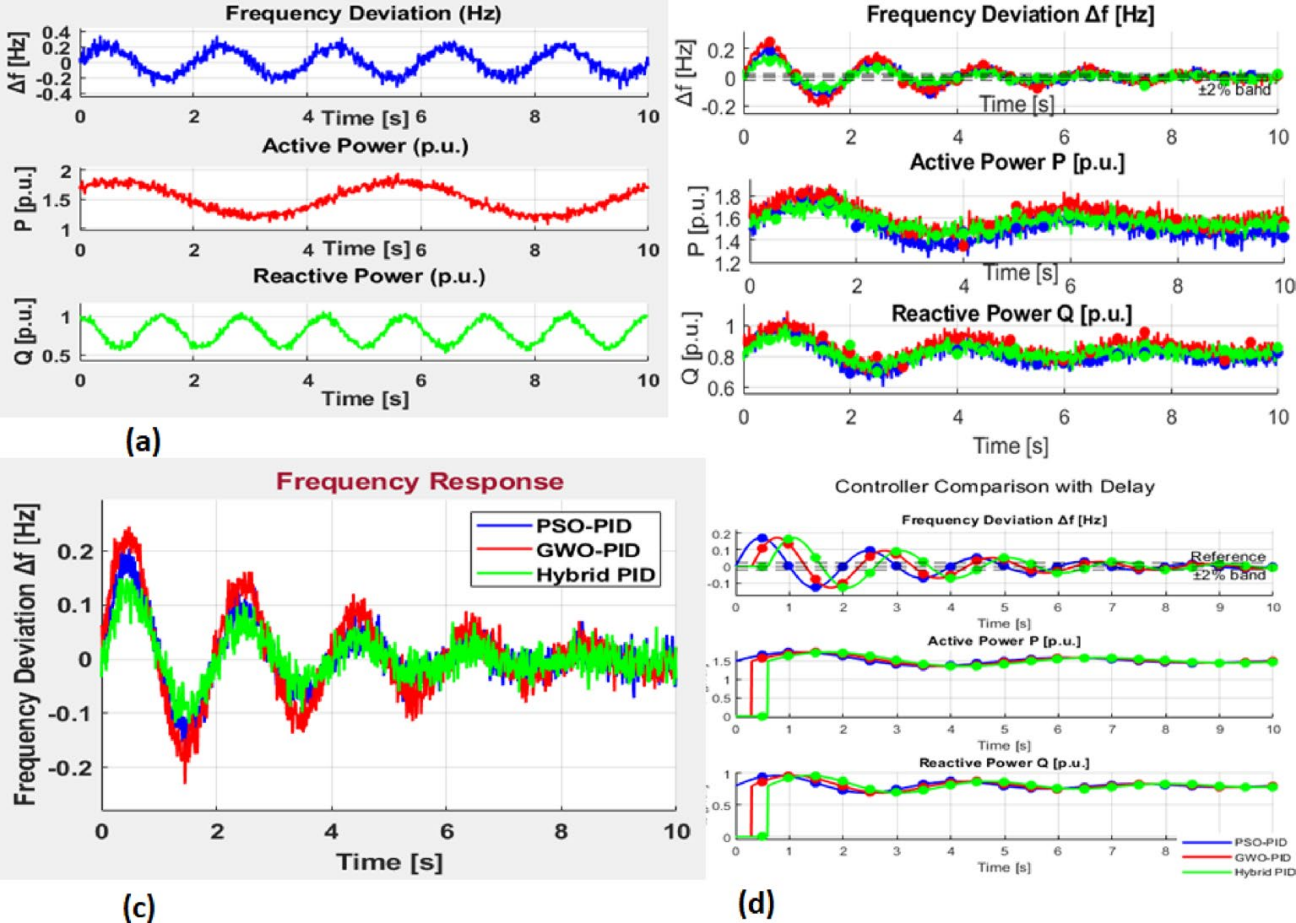
Real time results with OPAL-RT.

A significant variation in wind power is introduced as a disturbance to the MG, and the corresponding frequency deviation is evaluated in real time using the OPAL-RT. The frequency deviation under this disturbance, employing PSO, GWO, and hybrid PSO-GWO optimized PID controllers, is presented in Figs. 17b–c and 18. In addition, a continuous step change in wind power is applied to further assess the effectiveness of the three controllers, with the resulting frequency deviation illustrated in Fig. 17c. In Fig. 18a, the frequency deviation (Δf), active power (P in p.u.), and reactive power (Q in p.u.) responses clearly show that wind power fluctuations introduce noticeable oscillations in the system. Figure 18b compares the responses of different controllers, where the PSO-PID controller exhibits larger oscillations and slower damping. The GWO-PID controller improves the response with reduced oscillations but still demonstrates a moderate settling time. In contrast, the hybrid PSO-GWO based PID controller achieves the fastest damping and the smallest oscillations, maintaining the frequency within the acceptable ± 0.2 Hz band.

Figure 17c further illustrates that the PSO-PID controller has the highest overshoot and longest settling time, while the GWO-PID controller reduces overshoot but still exhibits noticeable oscillations. The hybrid PSO-GWO PID controller provides minimum peak deviation, faster settling time, and improved damping characteristics, confirming its superior capability in handling wind disturbances. Overall, analysis of both case studies indicates that the Hybrid PSO-GWO-PID controller significantly enhances frequency stability in terms of reduced peak overshoot and faster settling time compared to PSO-PID and GWO- PID controllers.

## Conclusion

This study demonstrates the effective application of SVR and DNN for wind speed prediction across four distinct geographic sites. The predictive performance of the models is evaluated using several statistical metrics, including MAE, Mean MAPE, MSE, RMSE, confidence interval, and standard deviation. The comparative analysis reveals that the SVR model consistently outperforms the DNN model, achieving significant reductions in prediction errors. SVR reduces MAE by up to 52.94%, MAPE by 44.59%, MSE by 83.54%, and RMSE by 59.55% across the considered sites, indicating its superior forecasting accuracy and robustness for wind speed prediction at four different locations for relatively smaller datasets (15 days). The proposed controller integrating SVR/DNN-based wind speed prediction with hybrid optimization techniques enhances frequency regulation in microgrids. However, its performance depends on the accuracy and generalization of the prediction models, which may vary across climatic conditions. Additionally, missing data can affect prediction reliability, and the combined use of wind prediction models with hybrid PSO-GWO optimization increases computational complexity, posing challenges for real-time implementation.

The wind speed predicted by the forecasting models is utilized as a disturbance input to the MG. To mitigate frequency deviations caused by wind power variability, a PID controller optimized using a hybrid PSO-GWO algorithm is proposed. Comprehensive time-domain and frequency-domain analyses, including Mp, Ts, and ISE, demonstrate the improved dynamic performance of the proposed hybrid controller compared with conventional PSO-PID and GWO-PID approaches. The results indicate that the PSO–GWO optimized PID controller significantly enhances the system performance over individual optimization methods. For the SVR-based wind prediction case, the hybrid PSO–GWO approach reduces Mp by 50%, Ts by 16.13%, and ISE by 77.78% compared to PSO, while achieving 33.33%, 10.86%, and 50% improvements over GWO, respectively. Similarly, for the DNN-based prediction case, the hybrid method provides even greater enhancements, reducing Mp by 92.5%, Ts by 21.21%, and ISE by 57.14% compared to PSO, confirming the superior effectiveness of the PSO–GWO optimization strategy. Furthermore, time-domain and frequency-domain analyses confirm the enhanced stability, reliability, and adaptability of renewable energy integrated MG systems. In addition, real-time validation using the OPAL-RT Hardware-in-the-Loop (HIL) platform verifies the practical applicability of the proposed controller for MG frequency regulation.

Additionally, Future work will focus on further enhancing the proposed framework by exploring advanced hybrid deep learning models to improve wind speed forecasting accuracy under varying climatic conditions. Further research may also investigate the application of advanced control strategies such as Robust Control, Model Predictive Control and other intelligent or adaptive controllers. Furthermore, the proposed approach can be extended to real MG implementations by considering nonlinear system characteristics, converter dynamics, and power electronic interfaces, enabling more realistic validation under practical operating conditions.

## Data Availability

The datasets generated and/or analyzed during the current study are not publicly available [solar and wind data collected from India Meteorological Department, Bhubaneswar are for research purpose. Need permission from IMD before making public] but are available from the corresponding author on reasonable request.

## Abbreviations

ANN: Artificial neural network
ARIMA: Auto-regressive integrated moving average
ARMA: Auto-regressive moving average
BESS: Battery energy storage system
DEG: Diesel engine generator
DL: Deep learning
DNN: Deep neural networks
FC: Fuel cell
FESS: Flywheel energy storage system
GWO: Grey wolf optimization
HHT-NAR: Hilbert–Huang transform
HIL: Hardware-in-the-Loop
ISE: Integral square error
KNN: K-nearest neighbors
LFC: Load frequency control
LSTM: Long short-term memory neural network
MAE: Mean absolute error
MG: Microgrid
Mp: Maximum peak overshoot
MSE: Mean square error
NAR: Nonlinear autoregressive neural network
NREL: National renewable energy laboratory
NWP: Numerical weather prediction
PID: Proportional integral derivative
PIs: Performance indices
PSO: Particle swarm optimization
RES: Renewable energy sources
RMSE: Root mean square
SVR: Support vector regression
SVM: Support vector machine
TF: Transfer function
Ts: Settling time
UC: Ultra capacitor
WES: Wind Energy systems
ΔMAPE: Mean Absolute percentage error
$$\Delta f$$: Frequency variation

## List of symbols

${G}_{FC}\left(s\right)$ ,${G}_{DEG}\left(s\right)$: Transfer Function of FC and DEG
${G}_{FESS}\left(s\right)$ ,${G}_{BESS}\left(s\right)$: Transfer Function of FESS and BESS
${G}_{WTG}(s)$,${G}_{UC}\left(s\right)$: Transfer Function of WTG and UC
|$${\dot{\mathrm{P}}}_{\mathrm{B}\mathrm{E}\mathrm{S}\mathrm{S}}$$|: Electromechanical constraints in BESS
${\dot{|\mathrm{P}}}_{\mathrm{D}\mathrm{E}\mathrm{G}}$|: Electromechanical constraints in DEG
|$${\dot{\mathrm{P}}}_{\mathrm{F}\mathrm{E}\mathrm{S}\mathrm{S}}$$|: Electromechanical constraints in FESS
${\dot{|\mathrm{P}}}_{\mathrm{U}\mathrm{C}}$|: Electromechanical constraints in UC
respt.: Respectively
